# A data compendium associating the genomes of 12,289 *Mycobacterium tuberculosis* isolates with quantitative resistance phenotypes to 13 antibiotics

**DOI:** 10.1371/journal.pbio.3001721

**Published:** 2022-08-09

**Authors:** The CRyPTIC Consortium

**Affiliations:** University of Oxford, Oxford, United Kingdom; Northern Arizona University, UNITED STATES

## Abstract

The Comprehensive Resistance Prediction for Tuberculosis: an International Consortium (CRyPTIC) presents here a data compendium of 12,289 *Mycobacterium tuberculosis* global clinical isolates, all of which have undergone whole-genome sequencing and have had their minimum inhibitory concentrations to 13 antitubercular drugs measured in a single assay. It is the largest matched phenotypic and genotypic dataset for *M*. *tuberculosis* to date. Here, we provide a summary detailing the breadth of data collected, along with a description of how the isolates were selected, collected, and uniformly processed in CRyPTIC partner laboratories across 23 countries. The compendium contains 6,814 isolates resistant to at least 1 drug, including 2,129 samples that fully satisfy the clinical definitions of rifampicin resistant (RR), multidrug resistant (MDR), pre-extensively drug resistant (pre-XDR), or extensively drug resistant (XDR). The data are enriched for rare resistance-associated variants, and the current limits of genotypic prediction of resistance status (sensitive/resistant) are presented by using a genetic mutation catalogue, along with the presence of suspected resistance-conferring mutations for isolates resistant to the newly introduced drugs bedaquiline, clofazimine, delamanid, and linezolid. Finally, a case study of rifampicin monoresistance demonstrates how this compendium could be used to advance our genetic understanding of rare resistance phenotypes. The data compendium is fully open source and it is hoped that it will facilitate and inspire future research for years to come.

## Abstract 2

This study by the CRyPTIC consortium presents an open-source dataset of 12,289 Mycobacterium tuberculosis clinical isolates from 23 countries on five continents. To facilitate faster and more targeted treatment of drug-resistant tuberculosis, the authors determined the genomic sequence and susceptibility profile for 13 drugs of each isolate.

## Introduction

Tuberculosis (TB) is a curable and preventable disease; 85% of those afflicted can be successfully treated with a 6-month regimen. Despite this, TB is the world’s top infectious disease killer (current SARS-CoV-2 pandemic excepted) with 10 million new cases and 1.2 million deaths estimated in 2019 alone [1]. Furthermore, drug-resistant TB (DR-TB; please see Table A in S1 File for a list of acronyms used throughout the manuscript) is a continual threat; almost half a million cases resistant to the first-line drug rifampicin (RR-TB) were estimated, with three-quarters of these estimated to be multidrug-resistant (MDR-TB, resistant to first-line drugs isoniazid and rifampicin) [1]. Worryingly, only 44% of DR-TB cases were officially notified and just over half of these cases were successfully treated (57%) [1].

To address these issues, the World Health Organisation (WHO) is encouraging the development of better, faster, and more targeted diagnostic and treatment strategies through its EndTB campaign [1,2]. Of particular interest is universal drug susceptibility testing (DST). Conventionally, DST relies on lengthy (4 weeks minimum) culture-based methods that require strict biosafety conditions for *Mycobacterium tuberculosis*. The development of rapid genetics-based assays has decreased diagnostic time to as little as 2 hours through the detection of specific resistance conferring mutations, e.g., the Cepheid Xpert MTB/RIF test [3,4]. However, assay bias towards specific genic regions can result in misdiagnosis of resistance, the prescription of ineffective treatment regimens, and subsequent spread of MDR disease, as seen during an MDR outbreak in Eswatini [5–7]. Furthermore, detection of rifampicin resistance is used to infer MDR-TB epidemiologically as rifampicin resistance tends to coincide with resistance to isoniazid [8]. While this *modus operandi* is successful at pragmatically identifying potential MDR cases quickly and effectively, it is not generally true that a single path exists for developing MDR or extensively drug resistant TB (XDR = MDR/RR + resistance to at least 1 fluoroquinolone and either bedaquiline or linezolid).

Whole-genome sequencing (WGS) has the potential to reveal the entirety of the *M*. *tuberculosis* genetic resistance landscape for any number of drugs simultaneously while enabling a more rapid turnaround time and reduction in cost compared to culture-based DST methods [9]. However, the success of WGS as a diagnostic tool wholly depends on there being a comprehensive and accurate catalogue of resistance-conferring mutations for each drug. Recent advances have shown that genotypic predictions of resistance correlate well with DST measurements for first-line drugs [8]. However, the mechanisms of resistance to second-line drugs along with the new and repurposed drugs (NRDs) are less well understood despite their increased administration in clinics as MDR cases climb [1,10].

To address these shortcomings, the Comprehensive Resistance Prediction for Tuberculosis: an International Consortium (CRyPTIC) has collected *M*. *tuberculosis* clinical isolates worldwide to survey the genetic variation associated with resistance to 13 antitubercular drugs, specifically the first-line drugs rifampicin, isoniazid, and ethambutol; the second-line drugs amikacin, kanamycin, rifabutin, levofloxacin, moxifloxacin, and ethionamide; and the NRDs bedaquiline, clofazimine, delamanid, and linezolid. Here, we introduce and describe these data in the form of an open-access data compendium of 12,289 isolates, each of which has had its genomic sequence determined and DST profile measured [11]. This compendium is the largest drug screening effort to date for *M*. *tuberculosis* in a “one isolate–one microscale assay” format across defined compound concentration ranges. A sampling process was designed to enrich for resistant isolates to account for the variable prevalence of resistance found in different countries and the many rare resistance mutations for several drugs. As a result, the compendium is not suitable for measuring prevalence, or estimating “real-world” error rates of resistance prediction tools; rather, it serves as a resource to accelerate antimicrobial resistance (AMR) diagnostic development by enriching mutation catalogues for WGS resistance prediction, improving our understanding of the genetic mechanisms of resistance, and identifying important diagnostic gaps and drug resistance patterns. Indeed, the consortium has begun to address some of these important issues in recent publications using this very compendium [12–16].

## Results

### The data compendium

**Fig 1:**
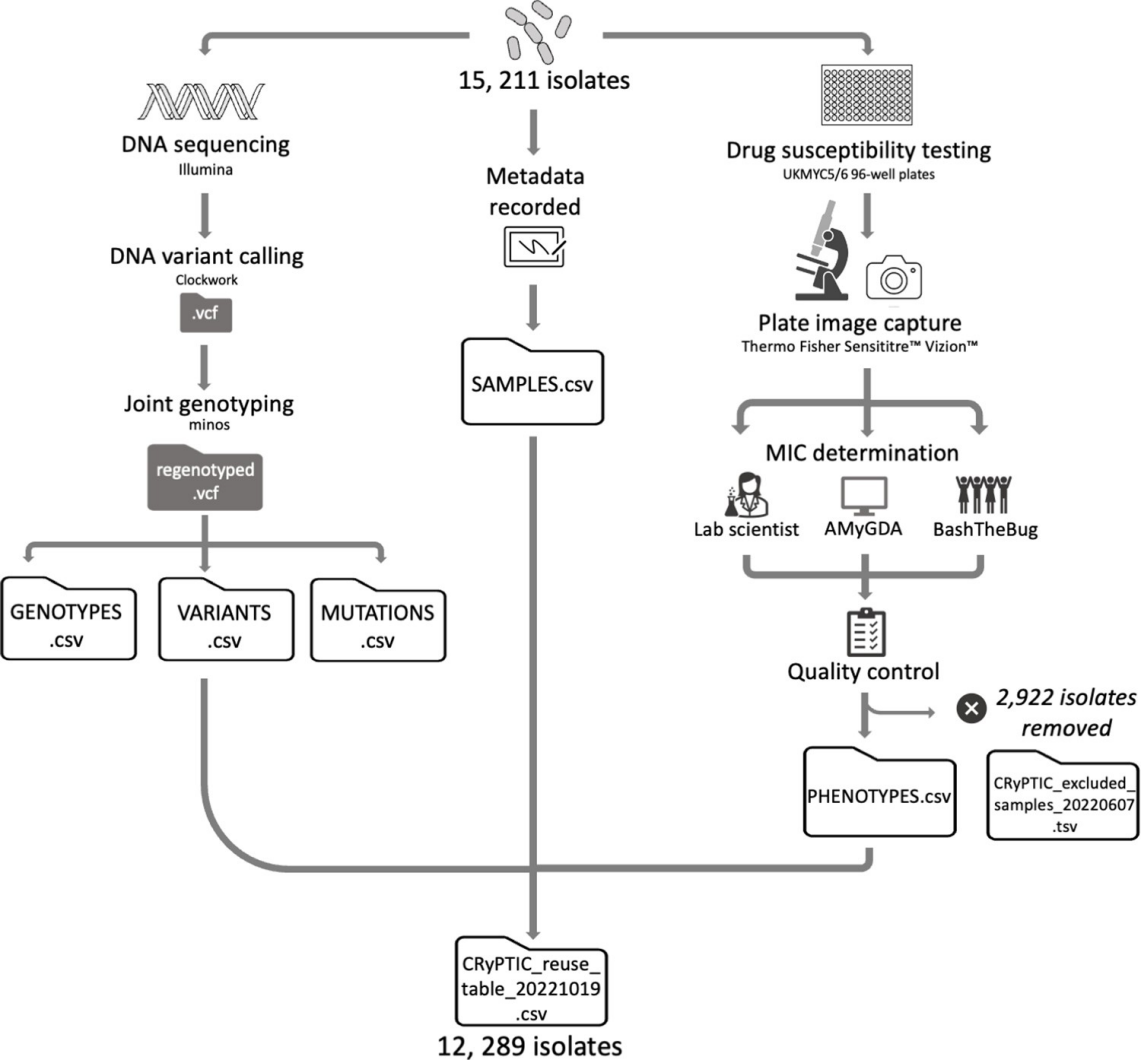
Processing sequencing and minimum inhibitory concentration data for 15,211 *Mycobacterium tuberculosis* isolates (“full” dataset). Briefly: Each isolate was DNA sequenced using an Illumina machine and plated onto 96-well plates (UKMYC5/6) containing 5–10× doubling dilutions of 13 antitubercular drugs for DST. Associated metadata (including country of origin and processing laboratory) was recorded. DNA variant calling and analysis was performed using Clockwork and Minos [47]). After 14 days, MIC measurements were taken by a trained scientist using Vizion, and the plate was photographed to also measure the MIC using the automated AMyGDA software and citizen scientists from BashTheBug [45]. After quality control procedures, phenotypic MIC data for 2,922 isolates were removed. The compendium therefore contains 15,211 isolates with WGS data (“full dataset”), 12,289 of which have matched quality assessed phenotypic data (“data compendium”). The raw sequence, VCFs, MICs, and binary resistance calls for the data compendium are presented in “CRyPTIC_reuse_table_20211019.csv” via an FTP site (see Methods), and the raw sequence and VCF files for those samples present in the full dataset are presented in “CRyPTIC_excluded_samples_20220607.tsv” via the same FTP site (see Methods). The data tables GENOTYPES.csv, VARIANTS.csv, MUTATIONS.csv, SAMPLES.csv, and PHENOTYPES.csv used for the analyses presented in this manuscript are also accessible via the FTP site (see Methods). DST, drug susceptibility testing; MIC, minimum inhibitory concentration; WGS, whole-genome sequencing.

The data compendium derives from a collection of 15,211 isolates for which both genomic and phenotypic data were collected by 23 of the partner countries across the continents of Asia, Africa, South America, and Europe. This collection will herein be referred to as the “full” dataset. An overview of the processing of the isolates is presented in Fig 1, and for a full description, please see Methods.

**Fig 2:**
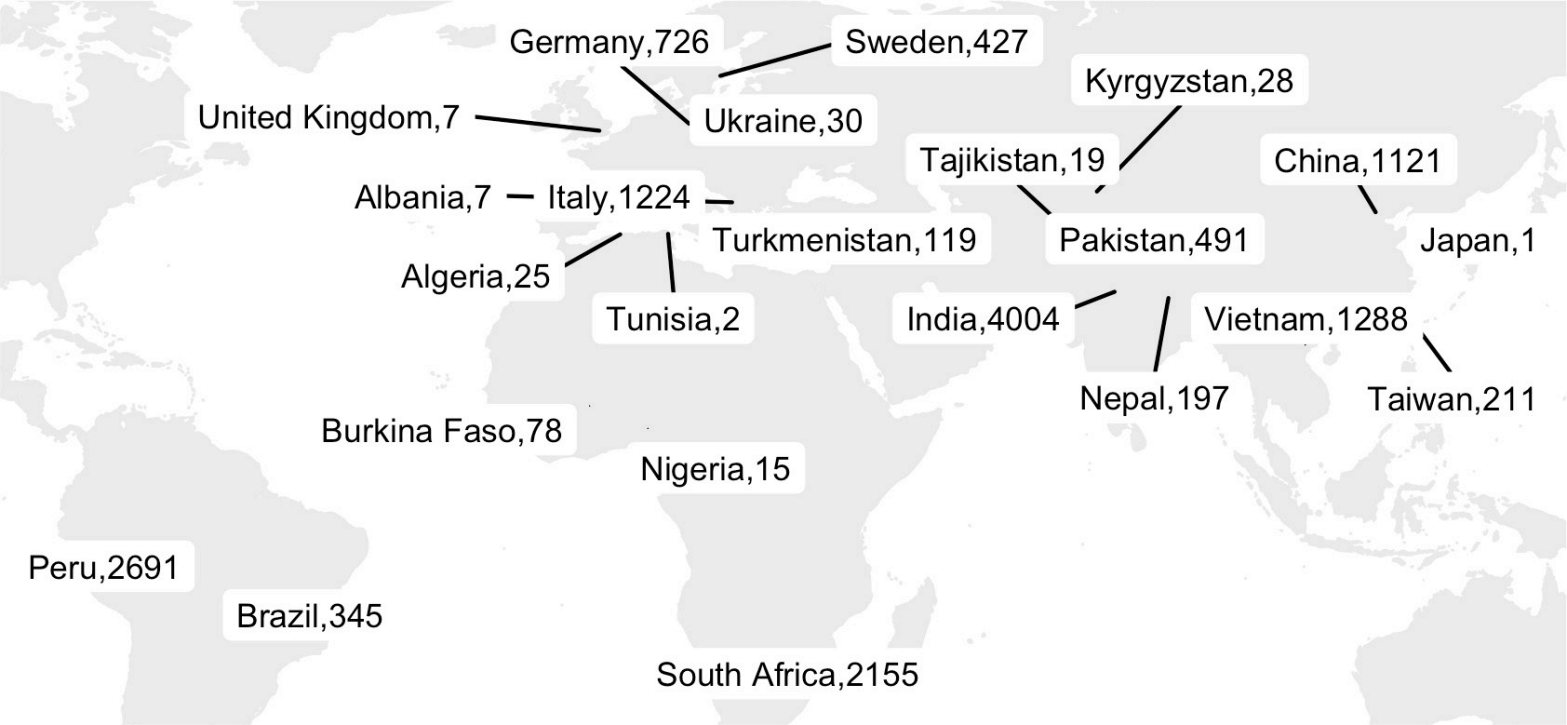
Geographical distribution of 15,211 CRyPTIC *Mycobacterium tuberculosis* clinical isolates (“full” dataset). The total number of isolates contributed by each country is depicted. Where the origin of an isolate was not known, the collection site identity was assigned (this occurred for 269 isolates in Germany, 17 isolates in India, 6 isolates in Peru, 885 isolates in Italy, 510 isolates in South Africa, 357 isolates in Sweden, 208 isolates in Taiwan, 1 isolate in Brazil, and 4 isolates in the UK). The base layer map was sourced from Natural Earth, which is in the public domain (see http://www.naturalearthdata.com/about/terms-of-use/), and created using the R packages “ggmap” and “maps” (see github.com/kerrimalone/Brankin_Malone_2022/).

The 15, 211 isolates originated from 23 different countries (Fig 2). The largest numbers of isolates were contributed by India (*n =* 4,004), Peru (*n* = 2,691), South Africa (*n* = 2,155), Vietnam (*n* = 1,288), and China (*n* = 1,121).

Lineage assignment revealed that 99.7% of the 15,211 isolates belong to the 4 main *M*. *tuberculosis* lineages (L1 to L4). The pie charts in Fig B in S1 File show the proportion of isolates among the different lineages (Table D in S1 File) and sublineages (Table E in S1 File) for each location. Like previous studies, we see a strong association between geolocation and lineage (Pearson’s chi-squared test, *p* < 2.2 × 10^−16^, Fig C in S1 File) [17,18]. The phylogenetic tree in Fig D in S1 File further highlights the strong population structure of this collection of isolates, with isolates clustering according to lineage. Typically undersampled in current databases and biobanks, the L3 isolates in this study represent the largest collection to date [19].

Although the 15,211 *M*. *tuberculosis* isolates belonging to the full dataset were plated to determine their minimum inhibitory concentrations (MICs) to 13 antitubercular drugs, regular quality assurance checks detected problems with plate inoculation and reading in 2 laboratories, resulting in the removal of 2,922 isolates. The data compendium therefore represents a total of 12,289 isolates with matched phenotypic and genotypic data for further analysis (Fig 1). Due to wells being skipped and other phenomena that prevent an MIC being measured, 88.1% of the isolates had a phenotype for all 13 drugs on the plate. For each drug, the number of isolates with an MIC measurement, and the associated quality of the reading, is presented in Table 1.

**Table 1:** Quality metrics for phenotype data.

	MIC measurements	HIGH QUALITY	MEDIUM QUALITY	LOW QUALITY
INH	12,070	9,519	1,351	1,200
RIF	12,099	8,955	1,356	1,788
EMB	12,158	7,506	1,355	3,297
LEV	12,163	7,774	1,354	3,035
MXF	12,194	6,785	1,353	4,056
AMI	12,072	8,973	1,350	1,749
KAN	12,130	9,333	1,355	1,442
BDQ	12,068	8,536	1,355	2,177
CFZ	12,049	7,763	1,352	2,934
DLM	11,927	8,095	1,349	2,483
LZD	12,189	7,141	1,355	3,693
ETH	12,132	8,821	1,355	1,956
RFB	12,150	10,042	1,352	756
TOTAL	**157,401**	**109,243**	**17,592**	**30,566**

Stated for each drug is the total number of MIC measurements stratified into “high” quality (at least 2 MIC measurement methods agree), “medium” quality (either Vizion and AMyGDA disagree, or the scientist recorded a MIC measurement using Vizion but did not store the plate picture), or “low” quality (all 3 MIC measurements methods disagree) phenotype classifications as described in Methods.

AMI, amikacin; BDQ, bedaquiline; CFZ, clofazimine; DLM, delamanid; EMB, ethambutol; ETH, ethionamide; INH, isoniazid; KAN, kanamycin; LEV, levofloxacin; LZD, linezolid; MXF, moxifloxacin; RFB, rifabutin; RIF, rifampicin.

### Resistance classification and distribution

**Fig 3:**
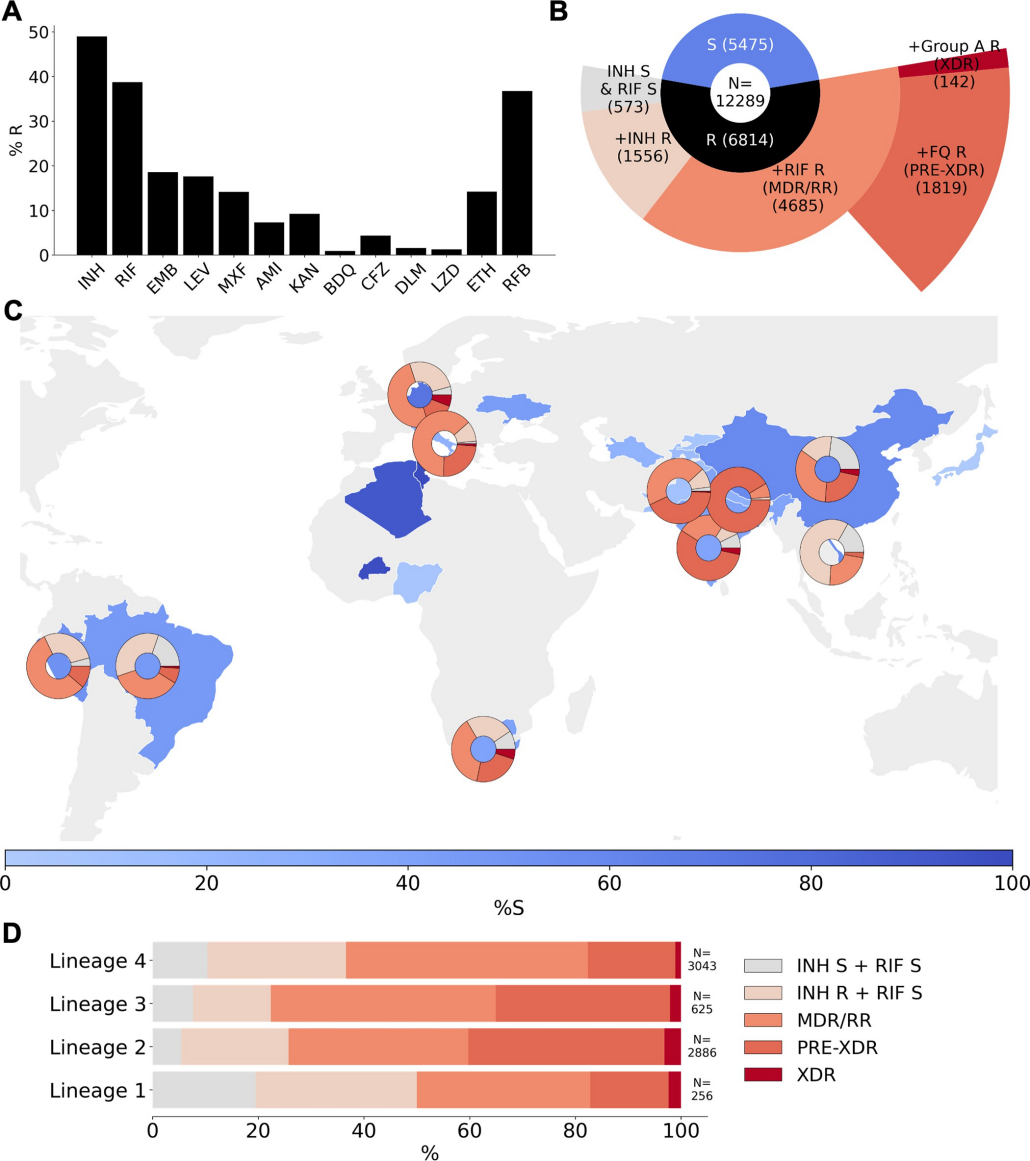
Drug phenotype data for the CRyPTIC compendium. **(A)** Frequency of resistance to each of 13 drugs in the data compendium. The total number of isolates with a binary phenotype (of any quality) for the corresponding drug is presented in Table 1. (**B)** Phenotypes of the 12,289 isolates with a binary phenotype for at least 1 drug. (**C)** Geographical distribution of phenotypes of 12,289 compendium isolates. Intensity of blue shows the percentage of isolates contributed that were categorised as susceptible to all 13 drugs (“%S”). Donut plots show the proportions of resistant phenotypes identified in (B) for countries contributing > = 100 isolates with drug resistance. **(D)** Proportions of resistance phenotypes in the 4 major *Mycobacterium tuberculosis* lineages. N is the number of isolates of the lineage called resistant to at least one of the 13 drugs. The base layer map in (C) was sourced from Natural Earth, which is in the public domain (see http://www.naturalearthdata.com/about/terms-of-use/), and created using the python library “geopandas” (see github.com/kerrimalone/Brankin_Malone_2022/). AMI, amikacin; BDQ, bedaquiline; CFZ, clofazimine; CRyPTIC, Comprehensive Resistance Prediction for Tuberculosis: an International Consortium; DLM, delamanid; EMB, ethambutol; ETH, ethionamide; INH, isoniazid; KAN, kanamycin; LEV, levofloxacin; LZD, linezolid; MDR, multidrug resistant; MXF, moxifloxacin; pre-XDR, pre-extensively drug resistant; RFB, rifabutin; RIF, rifampicin; RR, rifampicin resistant; XDR, extensively drug resistant.

Unsurprisingly, given its size and bias towards the collection of resistant isolates, resistance to each of the 13 drugs is represented within the compendium (Fig 3A). The drugs with the highest percentage of resistant isolates are the first-line drugs isoniazid and rifampicin (49.0% and 38.7%, respectively). Of the second-line drugs, levofloxacin had the highest proportion of resistant isolates in the dataset (17.6%) and amikacin the lowest (7.3%). A low proportion of isolates were resistant to the NRDs, bedaquiline (0.9%), clofazimine (4.4%), delamanid (1.6%), and linezolid (1.3%).

Of the 12,289 isolates represented by the compendium, 6,814 (55.4%) were resistant to at least 1 drug (Fig 3B). For the purpose of describing 5 broader resistance categories present in the dataset, we assumed that all MICs that could not be read had susceptible phenotypes. These 5 resistance categories comprise the following: isoniazid and rifampicin susceptible with resistance to another antitubercular drug, isoniazid resistant but rifampicin susceptible, RR/MDR, pre-XDR (RR/MDR + fluoroquinolone resistance), and XDR (RR/MDR + fluoroquinolone resistance + resistance to a group A agent: bedaquiline or linezolid). Consequently, the calculated prevalence of MDR, XDR, etc., in the dataset (Fig 3) are likely underestimates. Of the isolates resistant to one or more drugs, 22.8% were resistant to isoniazid and not rifampicin, 68.8% were either RR or MDR, and 8.4% were resistant to at least 1 antitubercular drug, but not isoniazid or rifampicin (Fig 3B). Of the RR/MDR isolates, 38.8% were pre-XDR and 3.0% were XDR. Two of the XDR isolates returned a resistant phenotype to all 13 of the drugs assayed (Table E in S1 File) and therefore could be reasonably described as totally drug resistant (TDR). One such isolate belonged to L4 and was contributed by South Africa, and the other belonged to L2 with an unknown country of origin contributed by Sweden.

The proportion of drug susceptible isolates collected differed between countries (Fig 3C, Methods). In countries that contributed more than 100 resistant isolates, each of the broad phenotypic resistance categories in Fig 3B were seen except for Peru, Vietnam, and Nepal, which did not contribute any XDR isolates (Fig 3C). Vietnam and Brazil sampled a high proportion of non-MDR/RR resistant phenotypes; 73.9% and 55.1% of resistant isolates contributed by these countries, respectively, were neither MDR nor RR. For Nepal and India, an especially high proportion of the MDR/RR isolates contributed were fluoroquinolone resistant (92.9% and 69.8%, respectively), which has been previously observed for this geographical region [1].

Of the 6,814 resistant isolates, 256 were from L1, 2,886 were from L2, 625 were from L3, and 3,043 were from L4. All 5 broader categories of resistance were represented in the 4 major *M*. *tuberculosis* lineages (Fig 3D). We note that the relative proportions of resistance categories will have been influenced by the different local sampling approaches since lineage distributions are typically geographically distinct (Fig B in S1 File). Bearing this in mind, we observe that in the compendium, L3 isolates contained the most MDR/RR isolates as a proportion of resistant isolates (77.6%), L2 isolates contained the most pre-XDR isolates as a proportion of MDR/RR isolates (54.2%), and L2 contained the most XDR isolates as a proportion of MDR/RR isolates (4.7%).

### Co-occurrence of drug resistance among the compendium isolates

**Fig 4:**
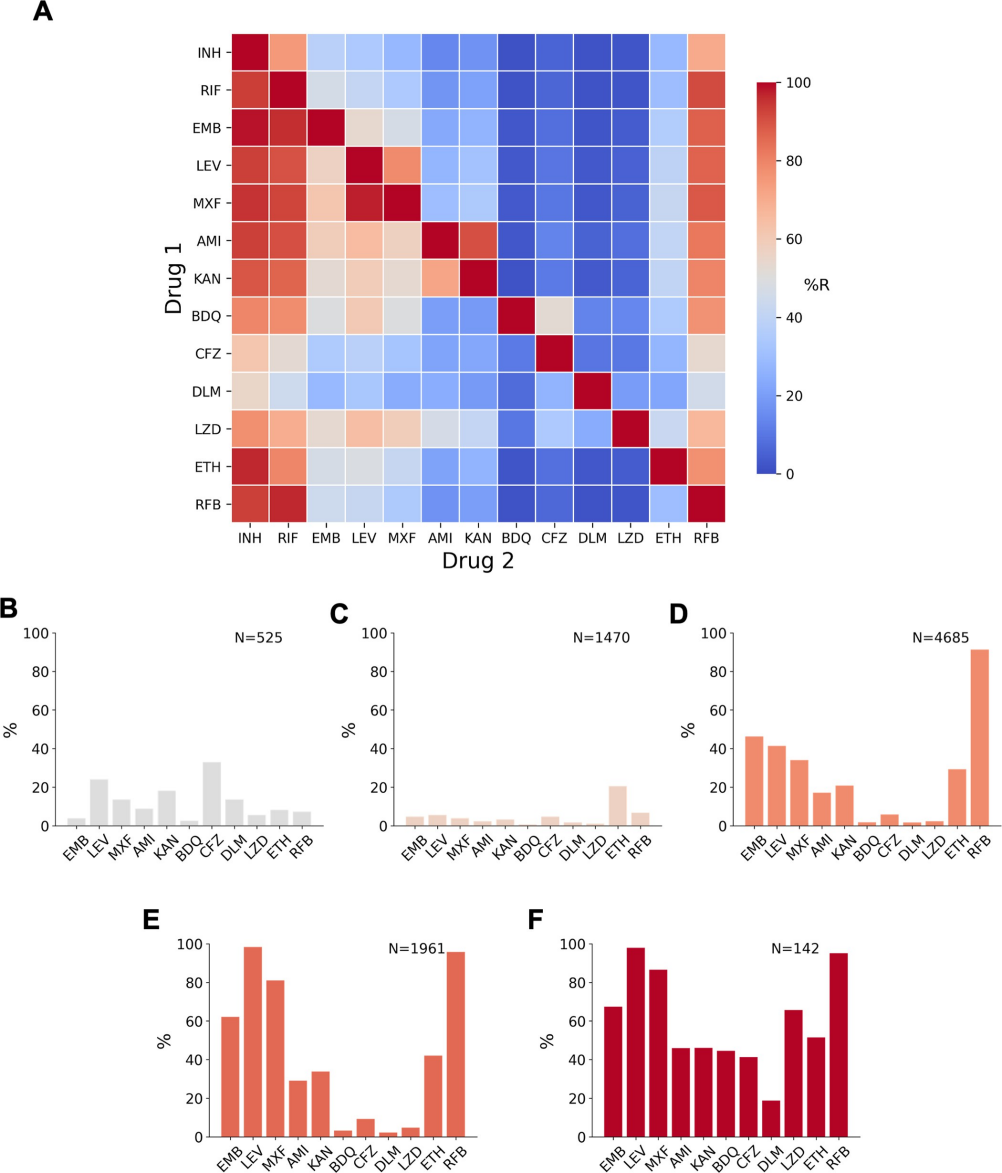
Co-occurrence of resistance to 1 drug conditional on resistance to another drug, or to resistance background. **(A)** The heatmap shows the probability of an isolate being resistant to Drug 2 if it is resistant to Drug 1, percentages are given in Table F in S1 File. **(B-F)** Percentage of isolates that are resistant to another of the 13 drugs in a background of **(B)** isoniazid susceptible + rifampicin susceptible (but resistant to at least one other antitubercular drug), **(C)** isoniazid resistant + rifampicin susceptible, **(D)** MDR/RR, **(E)** Pre-XDR, and **(F)** XDR. Only samples with definite phenotypes for RIF in MDR backgrounds and RIF and INH in non-MDR backgrounds and the additional drug are included. AMI, amikacin; BDQ, bedaquiline; CFZ, clofazimine; CRyPTIC, Comprehensive Resistance Prediction for Tuberculosis: an International Consortium; DLM, delamanid; EMB, ethambutol; ETH, ethionamide; INH, isoniazid; KAN, kanamycin; LEV, levofloxacin; LZD, linezolid; MDR, multidrug resistant (resistant to first-line drugs isoniazid and rifampicin); MXF, moxifloxacin; pre-XDR, pre-extensively drug resistant (MDR/RR + fluoroquinolone resistant); RFB, rifabutin; RIF, rifampicin; RR, rifampicin resistant; XDR, extensively drug resistant (MDR/RR + resistant to at least 1 fluoroquinolone and either bedaquiline or linezolid).

As we measured MICs to 13 drugs in parallel, we can ask whether, and how often, co-occurrence of drug resistance occurs among the isolates. We found isolates with all possible 2-drug resistant combinations in this dataset (Fig 4A and Table G in S1 File). With the exception of correlations between drugs in the same class (rifabutin versus rifampicin, moxifloxacin versus levofloxacin), isoniazid resistance was the most strongly associated with resistance to each of the other drugs. Resistance to any of the drugs was also strongly associated with resistance to rifampicin. Of the second-line drugs, levofloxacin and moxifloxacin were more commonly seen as a second resistant phenotype than the injectable drugs kanamycin and amikacin.

Resistance to both drugs in the aminoglycoside class was common in the dataset; 90.4% of amikacin-resistant isolates were also resistant to kanamycin, although significantly fewer kanamycin-resistant isolates were resistant to amikacin (72.0%, *p* < 0.00001) (Fig 4A). In a similar fashion, a smaller proportion of RR isolates were resistant to rifabutin than rifabutin-resistant isolates that were resistant to rifampicin (91.3%, 96.8%, *p* < 0.00001), while a smaller proportion of levofloxacin-resistant isolates were resistant to moxifloxacin than moxifloxacin-resistant isolates that were resistant to levofloxacin (78.5%, 97.6%, *p* < 0.00001). Differences in drugs of the same class are also well documented by in vitro studies [20–22].

Isolates resistant to the NRDs bedaquiline, clofazimine, delamanid, and linezolid were most likely to also be resistant to isoniazid, followed by rifampicin and rifabutin. The NRDs were less commonly seen as a second resistance phenotype and the smallest proportional resistance combinations involved the NRDs (e.g., 1.5% of isoniazid-resistant isolates were bedaquiline resistant). Within the NRDs, however, co-occurrence of resistance was proportionally higher; bedaquiline, linezolid, and delamanid resistance was commonly seen with clofazimine resistance (52.4%, 34.2%, and 26.3% of isolates having coresistance with clofazimine, respectively).

### Additional antibiotic resistance in isolates with non-MDR or MDR phenotypic backgrounds

To further investigate drug resistance patterns among the isolates, we examined in more detail the correlation structure of phenotypes by conditioning on different resistance backgrounds including isoniazid and rifampicin susceptible, isoniazid resistant and rifampicin susceptible, rifampicin resistant and isoniazid susceptible, MDR, pre-XDR, and XDR (Fig 4B–4F). We found that a greater proportion of isolates that were susceptible to isoniazid and rifampicin were resistant to the second-line drugs levofloxacin (24.1%), kanamycin (18.1%), moxifloxacin (13.7%), and amikacin (8.9%) than the first-line drug ethambutol (3.8%) (Fig 4B). The proportion of isolates resistant to clofazimine or levofloxacin was particularly high (32.9% and 24.1%, respectively), and more isolates were resistant to these 2 drugs than ethambutol in an isoniazid-resistant and rifampicin-susceptible background but not in MDR/RR isolates (Fig 4C–4F).

MDR/RR isolates were most commonly resistant (excluding rifabutin) to the first-line drug ethambutol (46.3%), closely followed by levofloxacin (41.4%). As expected, the proportion of fluoroquinolone resistance was higher in MDR/RR isolates than non-MDR isolates [23], and we found a greater proportion of isolates were resistant to levofloxacin than moxifloxacin, a pattern seen in all other backgrounds (Fig 4C–4F). For the aminoglycosides, a greater percentage of MDR/RR isolates were kanamycin resistant (21.8%) than amikacin resistant (18.1%), a trend seen in all other backgrounds.

For isolates with an XDR phenotype, a higher proportion were resistant to linezolid than bedaquiline (66.7% compared to 44.6%) and 11.3% of XDR isolates were resistant to both bedaquiline and linezolid (Fig 4F). XDR isolates were also resistant to the other NRDs, clofazimine (41.3%) and delamanid (18.8%). In non-XDR backgrounds, the most common NRD resistance seen was clofazimine (Fig 4B–4E).

### Genetic-based predictions of resistance

**Fig 5:**
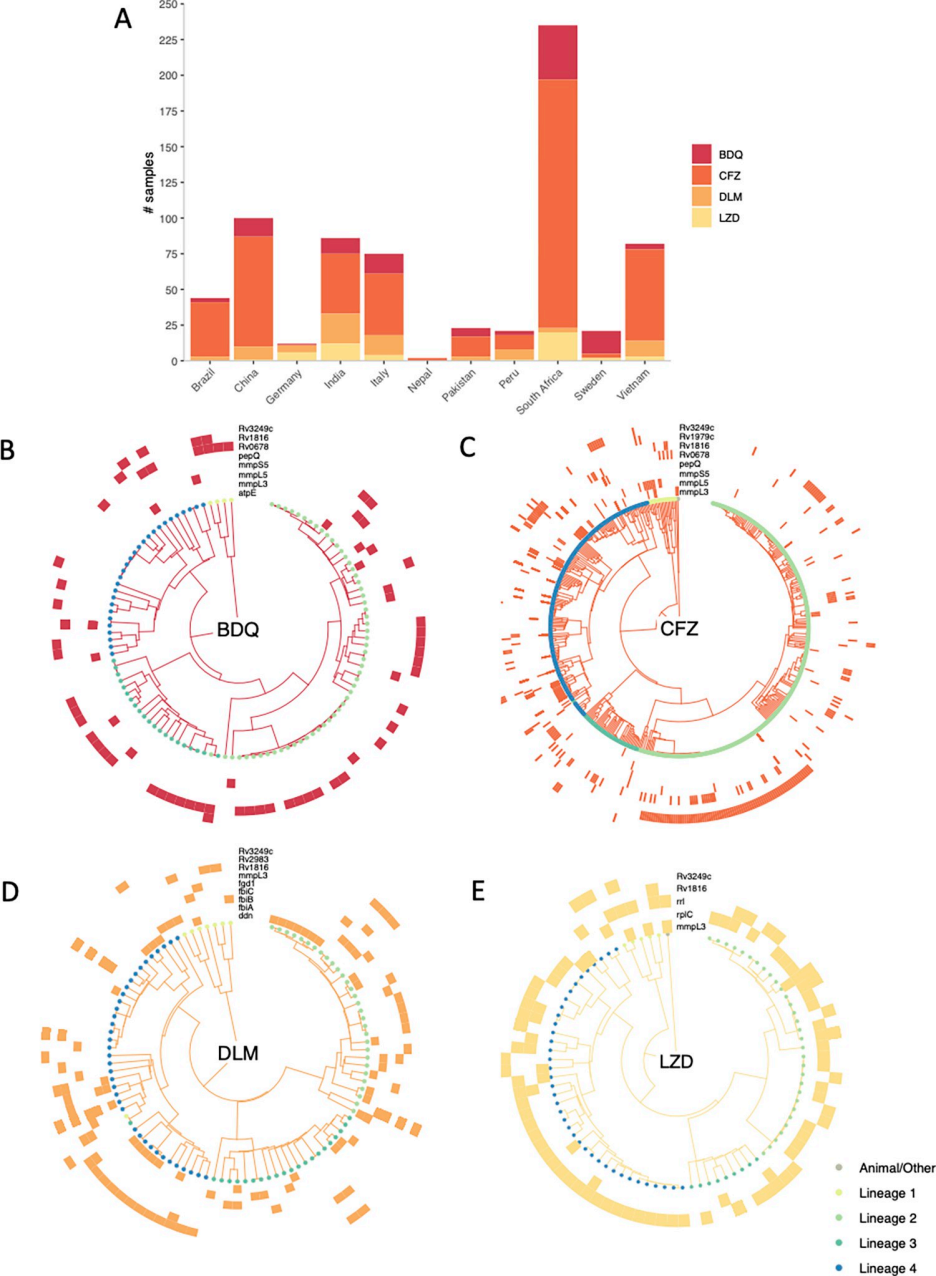
Resistance to bedaquiline, clofazimine, delamanid, and linezolid among *Mycobacterium tuberculosis* compendium isolates. **(A)** The prevalence (within these data) of resistance to BDQ, CFZ, DLM, and LZD per country or origin or collection site. Phylotrees are shown for isolates phenotypically resistant to **(B)** BDQ, **(C)** CFZ, **(D)** DLM, and **(E)** LZD. Tip point colours denote lineage. Each outer track represents a gene thought to be associated with resistance and coloured blocks denote the presence of a nonsynonymous mutation in the relevant gene for a given isolate. Mutations in these genes that are either associated with sensitivity or present in >5% of the collection of isolates as a whole were ignored. BDQ, bedaquiline; CFZ, clofazimine; DLM, delamanid; LZD, linezolid.

The compendium was sampled in order to collect as broad as possible a representation of resistance mutations and mechanisms—it is therefore expected to be enriched for rare resistance mutations compared with a prospectively sampled dataset from 1 country. To establish a baseline measure of how much unexplained resistance there was in the compendium, based on a standard catalogue of resistance mutations, we compared genetic-based predictions of susceptibility and resistance to the binary phenotypes derived from MICs for 8 drugs and for all isolates in this compendium (FN and VME columns in Table 2). Since these data are enriched for resistance, the calculated error rates are not representative of how well such a method would perform in routine clinical use. The results were broadly in line with prior measurements on a smaller (independent) set [24]. The hybrid catalogue used in this study does not make predictions for rifabutin, linezolid, bedaquiline, delamanid, or clofazimine; indeed, this is one of the main aims of the consortium and new catalogues published by CRyPTIC, and WHO will begin to address this shortcoming (Fig 5) [25].

**Table 2:** Predicting phenotypic resistance using genetics.

	TP	FP	TN	FN	VME	ME	PPV	NPV
**INH**	5,493	142	5,622	224	0.039	0.025	0.961	0.975
**RIF**	4,535	435	6,669	107	0.023	0.061	0.977	0.939
**EMB**	1,919	513	6,702	111	0.055	0.071	0.945	0.929
**LEV**	1,689	255	8,104	184	0.098	0.031	0.902	0.969
**MXF**	1,358	504	9,022	160	0.105	0.053	0.895	0.947
**AMI**	632	84	10,117	163	0.205	0.008	0.795	0.992
**KAN**	735	124	9,043	197	0.211	0.014	0.789	0.986
**ETH**	971	114	9,183	511	0.345	0.012	0.655	0.988

Statistics on how much resistance can be explained in a dataset enriched for rare resistance mutations using a standard resistance catalogue that predates the CRyPTIC project. TP, the number of phenotypically resistant samples that are correctly identified as resistant (“true positives”); FP, the number of phenotypically susceptible samples that are falsely identified as resistant (“false positives”); TN, the number of phenotypically susceptible samples that are correctly identified as susceptible (“true negatives”); FN, the number of phenotypically resistant samples that are incorrectly identified as susceptible (“false negative”); VME, very major error rate (false-negative rate), 0–1; ME, major error rate (false-positive rate), 0–1; PPV, positive predictive value, 0–1; NPV, negative predictive value, 0–1.

AMI, amikacin; EMB, ethambutol; ETH, ethionamide; INH, isoniazid; KAN, kanamycin; LEV, levofloxacin; MXF, moxifloxacin; RIF, rifampicin.

Table 3 shows the top mutations found among isolates phenotypically resistant to first- and second-line drugs. As expected, *rpoB* S450L was the most frequent mutation associated with rifampicin resistance, and *katG* S315T was the most common mutation associated with isoniazid resistance. Mutations in *gyrA* dominate among fluoroquinolone-resistant isolates; D94G and A90V are the 2 most frequently occurring mutations for levofloxacin and moxifloxacin.

**Table 3:** The top mutations associated with phenotypic drug resistance.

	GENE	VARIANT	n	%
**Rifampicin**	*rpoB*	S450L	2,914	62.2
	*rpoB*	D435V	506	10.8
	*rpoB*	H445D	202	4.3
	*rpoB*	H445Y	147	3.1
	*rpoB*	D435Y	112	2.4
**Isoniazid**	*katG*	S315T	3,668	70.0
	*fabG1*	c-15t	829	15.8
	*fabG1*	g-17t	176	3.4
	*fabG1*	t-8c	154	2.9
	*inhA*	I194T	56	1.1
**Ethambutol**	*embB*	M306V	1,131	50.0
	*embB*	M306I	1,001	44.3
	*embB*	Q497R	449	19.9
	*embB*	G406A	164	7.2
	*embB*	G406D	105	4.6
**Kanamycin**	*rrs*	a1401g	660	58.9
	*eis*	c-14t	70	6.2
	*eis*	g-10a	53	4.7
**Amikacin**	*rrs*	a1401g	660	74.7
	*rrs*	g1484t	7	0.8
**Levofloxacin**	*gyrA*	D94G	783	36.5
	*gyrA*	A90V	487	22.7
	*gyrA*	D94N	157	7.3
	*gyrA*	D94A	133	6.2
	*gyrA*	S91P	92	4.3
**Moxifloxacin**	*gyrA*	D94G	783	45.4
	*gyrA*	A90V	487	28.2
	*gyrA*	D94N	157	9.1
	*gyrA*	D94A	133	7.7
	*gyrA*	D94Y	70	4.1
**Ethionamide**	*fabG1*	c-15t	829	48.0
	*fabG1*	L203L	124	7.2

Depicted is a survey of the resistance-associated mutations present in the data compendium [8,26]. “VARIANT”: nonsynonymous amino acid mutations are denoted by upper case letters, while nucleotide substitutions for noncoding sequences are denoted by lower case letters. Negative numbers denote substitutions in promoter regions; “GENE”: genic region of interest in which “Variant” can be found; “*n*”: number of phenotypically resistant isolates with “VARIANT”; “%”: percentage of total phenotypically resistant isolates with “VARIANT”.

### Resistance to new and repurposed drugs

As previously stated, relatively few isolates are resistant to the NRDs, bedaquiline (*n* = 109), clofazimine (*n* = 525), delamanid (*n* = 186), and linezolid (*n* = 156). South Africa contributed the greatest number of isolates resistant to bedaquiline, clofazimine, and linezolid (Fig 5A), while China and India contributed the most isolates resistant to delamanid. Since the collection protocol differed between laboratories, it is not possible to infer any differences in the relative prevalence of resistance to the NRDs in these countries. The results of a survey of all nonsynonymous mutations in genes known or suspected to be involved in resistance to these 4 drugs (*rv0678*, *mmpL5*, *pepQ*, *ddn*, *rplC*, *rrl*, etc.) are depicted in Fig 5B–5E [27–31]. Mutations known to be associated with sensitivity were ignored, along with mutations that occurred at a frequency of ≥5% among all isolates (as 0.05% of total isolates are resistant to the NRDs). In contrast to first- and second-line drugs, there are no mutations within a single gene/small group of genes that can fully explain resistance to any NRD, and indeed no single gene (if there were, they would be visible as complete rings around the phylotrees in Fig 5). Note that the role of most of these mutations in resistance remains undetermined.

### Case study on rifampicin monoresistance

Around 1% of TB cases are rifampicin monoresistant (RMR), and the frequency is increasing [1,32]. The WHO does not recommend isoniazid for RMR treatment, despite it being effective; this is likely due to the reliance on assays such as Xpert MTB/RIF, which cannot distinguish between RMR and MDR. Use of isoniazid could improve treatment outcomes for RMR patients, which are currently similar to that of MDR TB, including a higher risk of death compared to drug-susceptible infections [33,34]. Due to its low natural prevalence, RMR has been poorly studied to date, but increasingly large clinical TB datasets, such as the one presented here, make its study now feasible.

**Fig 6:**
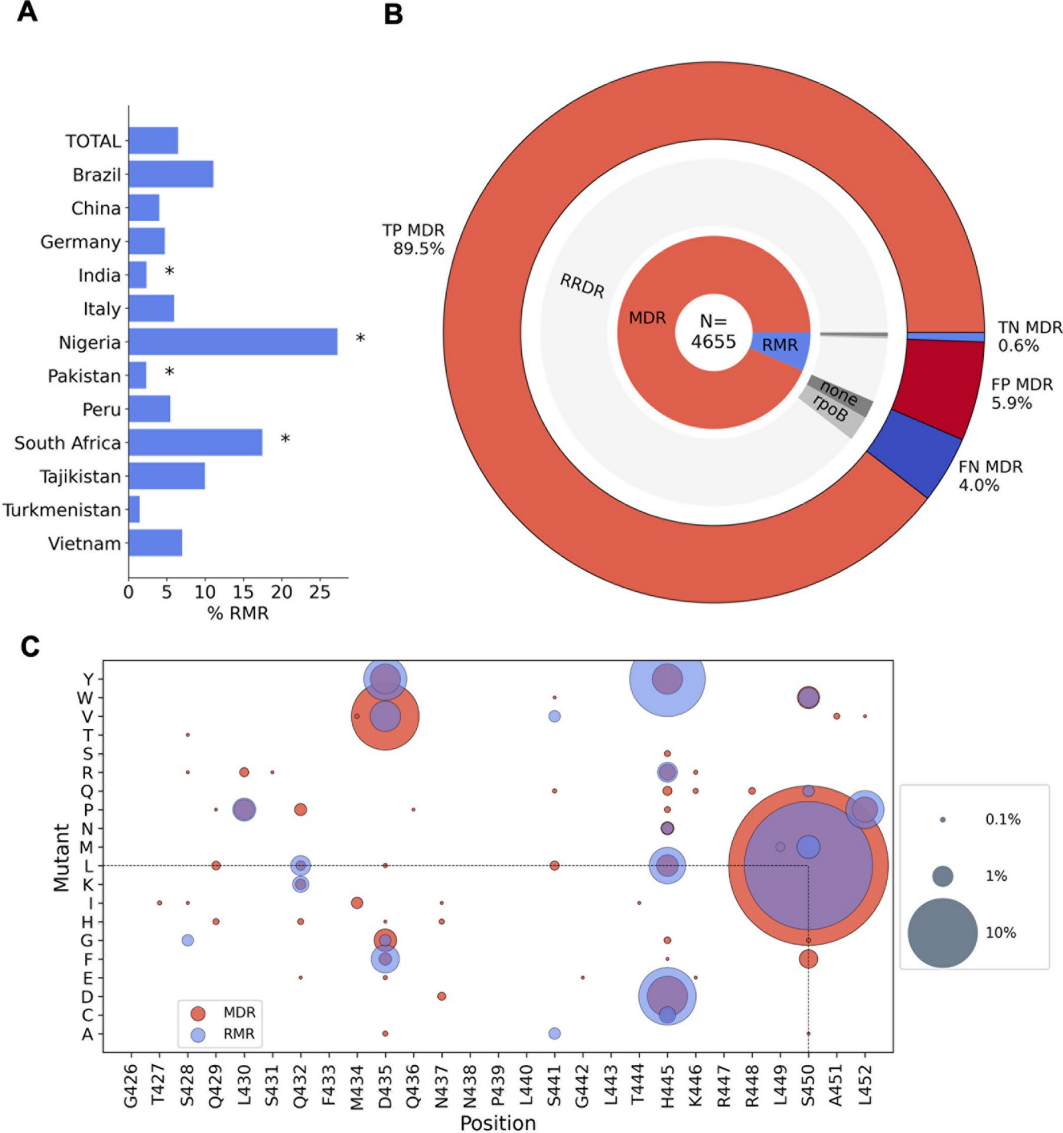
Rifampicin monoresistance. **(A)** Percentage of RR isolates that are RMR by country of isolate origin. * indicates RMR proportions that were significantly different from that of the total dataset using a 2-tailed z-test with 95% confidence. **(B)** MDR predictions for RR isolates made using the Xpert MTB/RIF assay proxy. N is the total number of RR isolates. The inner ring shows the proportion of RR isolates that are RMR and MDR. The middle ring represents the proportions of RMR and MDR isolates that have an SNP (synonymous or nonsynonymous) in the RRDR of *rpoB* (“RRDR”), no RRDR SNP but a SNP elsewhere in the *rpoB* gene (“*rpoB*”), and no *rpoB* mutations (“none”). The outer ring shows the expected TP, TN, FP, and FN MDR predictions of Xpert MTB/RIF assay, based on the SNPs present in the RR isolates. **(C)** Nonsynonymous mutations found in the RRDR of *rpoB* in RMR isolates and MDR isolates. Presence of a coloured spot indicates that the mutation was found in RMR/MDR isolates, and spot size corresponds to the proportion of RMR or MDR isolates carrying that mutation. FN, false negative; FP, false positive; MDR, multidrug resistant; RRDR, rifampicin resistance–determining region; RMR, rifampicin monoresistant; RR, rifampicin resistant; SNP, single nucleotide polymorphism; TN, true negative; TP, true positive.

For this case study, we defined RMR as any isolate that is rifampicin resistant and isoniazid susceptible, and discounted isolates with no definite phenotype for either drug. Of the 4,655 RR isolates in the compendium that also had a phenotype for isoniazid, 302 (6.5%) were RMR. These isolates were contributed by 12 different countries, and we found South African and Nigerian contributions had a significantly higher proportion of RMR isolates than that of the total dataset at 17.5% (*p* < 0.00001) and 27.3% (*p* = 0.00534), respectively (Fig 6A), compared with 6.5% for the total dataset. We note that these proportions are influenced by sampling strategies, but the higher contribution of RMR isolates from South Africa is consistent with previous studies [32].

### Rifampicin monoresistance is incorrectly predicted by current diagnostics

A widely used, WHO-endorsed diagnostic tool, the Xpert MTB/RIF assay, uses a proxy whereby any SNP detected in the “rifampicin-resistance determining region” (RRDR) of *rpoB* results in a prediction of MDR. However, the suitability of the proxy is dependent upon prevalence of RMR in the population [32]. We tested the reliability of this on the 4,655 RR isolates in our dataset that had a phenotype for isoniazid (Fig 6B).

Of these isolates, 4,353 (93.5%) were MDR and 302 (6.5%) were RMR. Of the MDR isolates, 187 had no RRDR mutation, and therefore, 4.0% of isolates in this study would be predicted as false negative MDR by the Xpert MTB/RIF assay. Of the RMR isolates, 276 had a mutation in the RRDR of *rpoB* and so the Xpert MTB/RIF assay proxy would incorrectly predict 5.9% of the RR isolates as false positive MDR cases. However, overall, the Xpert MTB/RIF assay proxy correctly predicts 89.5% of the RR isolates as MDR and 0.6% of the isolates as non-MDR in this dataset, which suggests it is a reasonably successful diagnostic tool with >90% accuracy for MDR classification of RR isolates. As our dataset is oversampled for resistance, it likely contains a higher prevalence of RMR than the global average, and, hence, the Xpert MTB/RIF assay is likely to perform better on more representative data. However, the analysis shows how the increasing global levels of RMR TB cases could increase the number of false positive MDR diagnoses by the Xpert MTB/RIF assay, denying isoniazid treatment to a greater number of patients who would then be moved on to less effective drugs.

### There are genetic differences between rifampicin monoresistant and multidrug resistant isolates

We have analysed the matched phenotypic and genotypic data of the compendium to examine whether there were any differences in the genetic determinants of rifampicin resistance between RMR and MDR isolates as was seen in a recent study of South African isolates [35]. The proportion of RMR isolates with no *rpoB* mutation (5.3%; Fig 6B) was significantly higher than that of MDR isolates (1.8%, *p* <0.00001). This suggests that non-target-mediated resistance mechanisms, such as up-regulation of rifampicin-specific efflux pumps, could be more influential in providing protection against rifampicin in RMR isolates than in MDR isolates.

The majority of RMR and MDR isolates contained one or more single nucleotide polymorphisms (SNPs) in *rpoB*, with the most having at least 1 mutation in the RRDR. To date, several nonsynonymous RRDR mutations have been found in RMR *M*. *tuberculosis* isolates, including the resistance conferring mutations S450L, H445D, and D435Y, which are also seen in MDR isolates [36,37]. For both RMR and MDR isolates in this dataset, the most common *rpoB* RRDR mutation seen was S450L (63.6% and 41.1% of isolates, respectively; Fig 6C). Five mutations were present in RMR isolates that were not seen in MDR isolates: S428G, S441A, S441V, S450M, and S450Q; however, these were seen at low prevalence (<2%) of RMR isolates. We found more RMR isolates had His445 mutated than MDR isolates (27.8% of RMR and 9.5% of MDR, *p* < 0.00001), and mutations at Ser450 and Asp435 were more prevalent in MDR isolates than RMR isolates (43.7% of RMR and 65.8% of MDR (*p* < 0.00001), and 9.3% of RMR and 15.5% of MDR (*p* = 0.00328), respectively).

In RMR isolates, we observed 27 different *rpoB* mutations that fall outside the RRDR; 11 were found in RMR but not MDR isolates and all were seen at <2% prevalence (Fig E in S1 File). The most common non-RRDR mutation in both MDR and RMR isolates was a cytosine to thymine mutation 61 bases upstream of the *rpoB* start codon (10.1% and 8.6% of isolates, respectively). The resistance conferring mutations, *rpoB* I491F, V695L, and V170F, were seen at low proportions (<2% of isolates) with no significant difference between MDR and RMR isolates.

## Discussion

This compendium of *M*. *tuberculosis* clinical isolates is the result of an extensive global effort by the CRyPTIC consortium to better map the genetic variation associated with drug resistance. Through its sheer size and by oversampling for resistance, the compendium gives an unparalleled view of resistance and resistance patterns among the panel of 13 antitubercular compounds studied. This study serves to summarise the data within the compendium and to highlight the existence of the open-access resource to the wider community to help better inform future treatment guidelines and steer the development of improved diagnostics.

Starting with first-line drugs, molecular based diagnostic assays have vastly improved the detection of and the speed at which we find DR-TB cases, resulting in improved quality of care for patients. However, relying solely on these diagnostic methods has several drawbacks. Aside from the Xpert MTB/RIF assay potentially increasing false positive MDR diagnoses as discussed earlier in the RMR case study, the assay assumes isoniazid resistance upon detection of rifampicin resistance. Thus, less is known about the prevalence of mono-isoniazid resistance or “true” cases of MDR (confirmed rifampicin and isoniazid resistance) [1] and with large datasets such as this compendium, we can further investigate these important and rarer clinical phenotypes (like that of RMR in our case study). Another example of a rarer phenotype is that of isoniazid-resistant and rifampicin-susceptible (Hr-TB) isolates; a greater number of these were contributed by CRyPTIC countries than RMR isolates *(n =* 1,470 versus *n* = 302), a pattern also recently observed in a global prevalence study [38]. A modified 6-month treatment regimen is now recommended for Hr-TB (rifampicin, ethambutol, levofloxacin, and pyrazinamide), and as a result of inadequate diagnosis, many of the 1.4 million global Hr-TB estimated cases would have received inadequate and unnecessarily longer treatment regimens [1,39]. Encouragingly, CRyPTIC isolates with an Hr-TB background exhibited relatively low levels of resistance to other antitubercular drugs, including those in the augmented regimen (Fig 4C). However, without appropriate tools to assess and survey this, we will continue to misdiagnose and ineffectively treat these clinical cases. In 2018, CRyPTIC and the 100,000 Genomes project demonstrated that genotypic prediction from WGS correlates well with culture-based phenotype for first-line drugs, which is reflected in our summary of the genetic catalogue applied to this dataset (Table 3) [8]. While predictions can be made to a high level of sensitivity and specificity, there is still more to learn, as exemplified by the isolates in the compendium that despite being resistant to rifampicin and isoniazid could not be described genetically (Table 2). This shortfall, along with the limitations of molecular based diagnostic assays, highlights the need for continual genetic surveillance and shines a favourable light on a WGS-led approach.

A strength of this compendium lies with the data collated for second-line drugs. A greater proportion of drug-resistant isolates had additional resistance to fluoroquinolones than second-line injectable drugs (Fig 4A). This could be due to more widespread use of fluoroquinolones as well as their ease of administration and hence them being recommended over injectables for longer MDR treatment regimens [1]. Concerningly, we found that resistance to levofloxacin and moxifloxacin, and kanamycin and amikacin, were more common than resistance to the mycobacterial specific drug ethambutol in an isoniazid- and rifampicin-susceptible background (Fig 4B), suggesting a level of preexisting resistance to second-line drugs. This concurs with a systematic review that found patients previously prescribed fluoroquinolones were 3 times more likely to have fluoroquinolone-resistant TB [40]. Careful stewardship of fluoroquinolones, both in TB and other infectious diseases, will be paramount for the success of treatment regimens. Despite variability in sample collection, we observed high proportions of fluoroquinolone-resistant MDR/RR isolates from some countries and therefore suggest that MDR treatment regimens could be improved by optimisation on a geographic basis.

Further treatment improvement could also be made by the selection of appropriate drugs from each class. For example, The WHO recommends switching from kanamycin to amikacin when treating MDR TB patients [39], and the compendium supports this recommendation as we saw more resistance to kanamycin than amikacin in all phenotypic backgrounds. For fluoroquinolones, more isolates were resistant to levofloxacin than moxifloxacin in all phenotypic backgrounds, suggesting moxifloxacin may by the most appropriate fluoroquinolone to recommend, although we note this conclusion is critically dependent on the validity of the cutoff, here an ECOFF, used to infer resistance. However, the amenability of drugs to catalogue-based genetic diagnostics is also an important consideration, and our data suggest levofloxacin resistance could be predicted more reliably than moxifloxacin, with fewer false positives predicted (Table 2). Testing for fluoroquinolone resistance using molecular diagnostic tests remains limited. Global data from the past 15 years suggest that the proportion of MDR/RR TB cases resistant to fluoroquinolones sits at around 20%, with these cases primarily found in regions of high MDR-TB burden [1]. While recently approved tools, such as the Cepheid Xpert MTB/XDR cartridge, will permit both isoniazid and fluoroquinolone testing to be increased, the same pitfalls are to be encountered regarding targeted diagnostic assays [41]. In contrast, the genetic survey in this study demonstrates the potential of WGS for genetic prediction of resistance to second-line drugs, and studies within the consortium to investigate this are underway.

The data compendium has facilitated the first global survey of resistance to NRDs. Reassuringly, prevalence of resistance to the NRDs was substantially lower than for first- and second-line agents in the dataset (Fig 3A), and resistance to the new drugs bedaquiline and delamanid was less common than the repurposed drugs clofazimine and linezolid in an MDR/RR background (Fig 4C). However, the presence of higher levels of delamanid and clofazimine resistance than ethambutol resistance in the isoniazid- and rifampicin-susceptible background does suggest some preexisting propensity towards NRD resistance (Fig 4B).

Coresistance between NRDs was seen in isolates in the compendium, the most common being isolates resistant to both bedaquiline and clofazimine. This link is well documented and has been attributed to shared resistance mechanisms such as nonsynonymous mutations in *rv0678*, which were found in both clofazimine- and bedaquiline-resistant isolates in the compendium [31] (Fig 5B and 5C). Increased clofazimine use could further increase the prevalence of *M*. *tuberculosis* isolates with clofazimine and bedaquiline coresistance, limiting MDR treatment options including using bedaquiline as the backbone of a shorter MDR regimen [42]. Therefore, proposed usage of clofazimine for other infectious diseases should be carefully considered.

WHO recommends against the use of bedaquiline and delamanid in combination to prevent the development of coresistance, which could occur relatively quickly [43]; the rate of spontaneous evolution of delamanid resistance *in vitro* has been shown to be comparable to that of isoniazid, and, likewise, bedaquiline resistance arises at a comparable rate to rifampicin resistance [44]. In this compendium, 12.9% of bedaquiline-resistant isolates were resistant to delamanid, and 7.1% of delamanid-resistant isolates were resistant to bedaquiline. Several scenarios could account for this, including the presence of shared resistance mechanisms. For example, as bedaquiline targets energy metabolism within the cell, changes to cope with energy/nutrient imbalances upon the acquisition of resistance-associated ATPase pump mutations may result in cross resistance to delamanid in a yet unknown or unexplored mechanism [13]. It is imperative that genetic determinants of resistance are fully explored for the NRDs, as these are our current treatments of last resort, with special attention given to those mechanisms that could be shared with other agents. In the meantime, careful stewardship and phenotypic and genotypic surveillance of the NRDs should be implemented, including linezolid and clofazimine, which are now group A and B drugs, respectively, for MDR treatment [1].

Several research avenues are being actively explored by the CRyPTIC consortium that make further use of this compendium, including the following: (i) relating genetic mutations to quantitative changes in the MICs of different drugs [13]; (ii) genome-wide association studies [15]; (iii) training machine learning models that can predict resistance [14]; and (iv) exploration of the genetic determinants of resistance to second line and NRDs [16]. Collectively, these studies share the same aim of facilitating the implementation of WGS-directed resistance prediction in the clinic. Finally, we urge other researchers to explore and analyse this large dataset of *M*. *tuberculosis* clinical isolates and hope it will lead to a wave of new and inciteful studies that will positively serve the TB community for years to come.

## Methods

### Ethics

Approval for CRyPTIC study was obtained by Taiwan Centers for Disease Control IRB No. 106209, University of KwaZulu Natal Biomedical Research Ethics Committee (UKZN BREC) (reference BE022/13) and University of Liverpool Central University Research Ethics Committees (reference 2286), Institutional Research Ethics Committee (IREC) of The Foundation for Medical Research, Mumbai (Ref nos. FMR/IEC/TB/01a/2015 and FMR/IEC/TB/01b/2015), Institutional Review Board of P.D. Hinduja Hospital and Medical Research Centre, Mumbai (Ref no. 915-15-CR [MRC]), scientific committee of the Adolfo Lutz Institute (CTC-IAL 47-J / 2017) and in the Ethics Committee (CAAE: 81452517.1.0000.0059) and Ethics Committee review by Universidad Peruana Cayetano Heredia (Lima, Peru) and LSHTM (London, UK), Institutional Review Board at Pham Ngoc Thach Hospital, HCMC, Vietnam and Oxford Tropical Research Ethics Committee, UK, University of the Witwatersrand, Johannesburg Human Research Ethics Committee (Medical) (M160667), Technical Scientific Council (CTC-IAL no. 47-J / 2017) and Research Ethics Committee (CAAE 81452517.1.0000.0059) of Adolfo Lutz Institute, and University off Cape Town Faculty of Health Sciences Research Ethical Committee approvals (HREC 012/2007, 057/2013).

The CRyPTIC study involves analysis of microbiological isolates only—there is no associated data on patients. The study aggregates isolates from previous studies (which had previously obtained IRB approval) and also collected its own samples. Each IRB listed above assessed the protocol and saw no need for individual consent as only microbiological isolates were being analysed, and no personally identifiable information or host genetic data was used. In the remaining jurisdictions (IML Gauting (Germany), Public Health Scotland, Public Health Sweden, San Raffaele Scientific Institute, Italy), no IRB approval (and no individual patient consent) was required for studies analysing routinely collected microbiological isolates only.

### Sample collection

This study was designed to identify as many drug resistance mechanisms and mutations as possible. Given that for many drugs there is a long tail of rare mutations present at different frequencies in different countries, sample collection was biased towards collecting resistant isolates, with (wherever possible) temporally and geographically matched susceptibles. Therefore, with 4 exceptions, all collecting sites either sequenced all culturable isolates, a random subsample of all culturable isolates (tailored to budget) or sequenced a subsample of all resistant with matched susceptible samples. The exceptions were as follows: 102 samples from South Africa, which were a clinical cohort recruited on the basis of the health service classifying them as RR; the first 1,000 out of 2,944 samples from Peru were a historical freezer collection with heterogeneous sampling process (and the remainder were sampled prospectively, at random); Brazilian samples combined all stored resistant samples, with prospectively sampled pan-susceptibles; in addition to sampling prospectively and retrospectively (from freezers) enriching for resistance and matched susceptibles, at IML Gauting (Germany) and National Institute for Communicable Diseases (Johannesburg), all isolates resistant to a NRD were included. Chinese isolates were collected according to the following strategy: collecting subsites were randomly selected from the 72 counties, and then all culture-positive isolates were included. A broad breakdown of sampling approaches is included in Table B in S1 File.

### Plate assay

The CRyPTIC consortium designed 2 versions of the Sensititre MYCOTB plate (Thermo Fisher Scientific, USA) named the “UKMYC5” and “UKMYC6” microtitre plates [11,12]. These plates contain 5 to 10 doubling dilutions of 13 antibiotics (rifampicin (RIF), rifabutin (RFB), isoniazid (INH), ethambutol (EMB), levofloxacin (LEV), moxifloxacin (MXF), amikacin (AMI), kanamycin (KAN), ethionamide (ETH), clofazimine (CFZ), linezolid (LZD), delamanid (DLM), and bedaquiline (BDQ)). DLM and BDQ were provided by Otsuka Pharmaceutical and Janssen Pharmaceuticals, respectively. The UKMYC5 plate also contained para-aminosalicylic acid (PAS), but the MICs were not reproducible, and, hence, it was excluded from the UKMYC6 plate design and is not included in any subsequent analysis [11].

A standard operating protocol for sample processing was defined by CRyPTIC as previously described [11,12]. Clinical samples were subcultured using 7H10 agar plates, Lowenstein–Jensen tubes, or MGIT tubes. Bacterial cell suspensions (0.5 McFarland standard, saline Tween) prepared from (no later than) 14-day-old colonies were diluted 100X in 10 ml enriched 7H9 broth prior to plate inoculation. A semiautomated Sensititre Autoinoculator (Thermo Fisher Scientific, USA) was used to inoculate 100 μl prepared cell suspensions (1.5 × 10^5^ CFU/ml [5 × 10^4^ CFU/ml—5 × 10^5^ CFU/ml]) into each well of a UKMYC5/6 microdilution plate. The plate was sealed and incubated for 14 days at 37°C. Quality control runs were performed periodically using *M*. *tuberculosis* H37Rv ATCC 27294, which is sensitive to all drugs on the plates.

### Minimum inhibitory concentration (MIC) measurements

MICs for each drug were read after incubation for 14 days by a laboratory scientist using a Thermo Fisher Sensititre Vizion digital MIC viewing system [11]. The Vizion apparatus was also used to take a high contrast photograph of the plate with a white background, from which the MIC was measured again using the Automated Mycobacterial Growth Detection Algorithm (AMyGDA) software [45]. The AMyGDA algorithm was specifically developed to automate and perform quality control of MIC measurements and to facilitate machine learning studies within the consortium. AMyGDA detects the boundaries of each well using a Hough transform for circles and measures growth as the number of dark pixels within the area contained by this boundary.

All images where the MICs measured by Vizion and AMyGDA were different were uploaded to a citizen science project, BashTheBug, on the Zooniverse platform [46]. Each image was then classified by ≥11 volunteers and the median classification taken. MICs were then classified as high (at least 2 methods concur on the MIC), medium (either a scientist recorded a MIC measurement using Vizion but did not store the plate picture, or Vizion and AMyGDA disagree and there is no BashTheBug measurement), or low (all 3 methods disagree) quality.

To ensure adequate data coverage for this study, we took the MIC from the Vizion reading provided by the trained laboratory scientist if it was annotated as having medium or low quality.

### Binary phenotype classification

Binary phenotypes (resistant/susceptible) were assigned from the MICs by applying epidemiological cutoff (ECOFF) values [12]; samples with MICs at or below the ECOFF are, by definition, wild-type and hence assigned to be susceptible to the drug in question [12]. Samples with MICs above the ECOFF are therefore classified as resistant (Fig A and Table C in S1 File). Please see [12] for the body of work supporting the use of the ECOFF relative to the compendium isolates and Table C in S1 File for the ECOFFs for each drug tested.

### Genomic data processing and variant calling

Clinical samples were subcultured either using Lowenstein–Jensen tubes, 7H10 agar plates, or MGIT tubes for (no more than) 14 days prior to DNA extraction using either the FastPrep-24 instrument (MP Biomedicals) for cell lysis and ethanol precipitation or the cetyltrimethylammonium bromide (CTAB) method. Paired-end libraries were prepared using a Nextera XT DNA sample preparation kit (Illumina, San Diego, CA, USA) and were sequenced on Illumina instruments. The resulting FASTQ files were processed using the bespoke pipeline Clockwork (v0.8.3, github.com/iqbal-lab-org/clockwork; [47]). Briefly, all raw sequencing files were indexed into a relational database with which Clockwork proceeds. Human, nasopharyngeal flora, and human immunodeficiency virus–related reads were removed, and remaining reads were trimmed (adapters and low-quality ends) using Trimmomatic and mapped with BWA-MEM to the *M*. *tuberculosis* H37Rv reference genome (NC000962.3) [48,49]. Read duplicates were removed. Genetic variants were called independently using Cortex and SAMtools, 2 variant callers with orthogonal strengths (SAMtools, a high-sensitivity SNP caller, and Cortex, a high-specificity SNP and indel caller) [50,51]. The 2 call sets were merged to produce a final call set, using the Minos adjudication tool (v0.11.0) to resolve loci where the 2 callers disagreed, by remapping reads to an augmented genome containing each alternative allele [24]. Default filters of a minimum depth of 5×, a fraction of supporting reads of 0.9 (Minos) and a genotype confidence percentile (GCP) filter of 0.5 were applied. The GCP filter is a normalised likelihood ratio test, giving a measure of confidence in the called allele compared with the other alternatives, and is described in [24]. This produced one variant call format (VCF) file per sample, each only describing positions where that sample differed from the reference.

These filtered VCFs were then combined to produce a single nonredundant list of all variants seen in the cohort. All samples were then processed a second time with Minos, remapping reads to a graphical representation of all the segregating variation within the cohort, generating VCF files that had an entry at all variable positions (thus for all samples, most positions would be genotyped as having the reference allele). These “regenotyped” VCFs were later used to calculate pairwise distances (see below). Please refer to Supplemental Method A in S1 File for commands used to generate the per-sample and regenotyped VCF files.

To remove untrustworthy loci, a genome mask was applied to the resulting VCF files (regions identified with self-blast matches in [52] comprising of 324,971 bp of the reference genome). Furthermore, positions with less than 90% of total samples passing default Clockwork/Minos variant call filters (described above) were filtered out, comprising 95,703 bp of the genome, of which 55,980 bp intersect with the genome mask.

### Resistance prediction using a genetic catalogue

A hybrid catalogue of genetic variants associated with resistance to first- and second-line drugs based on existing catalogues was created and can be found at github.com/oxfordmmm/tuberculosis_amr_catalogues/blob/public/catalogues/NC_000962.3/NC_000962.3_CRyPTIC_v1.311_GARC1_RUS.csv [8,26]. We specifically did not use the recent WHO catalogue to avoid circularity and overtraining, as that catalogue was developed (via prior literature, expert rules, and a heuristic algorithm) based partially on these isolates [25]).

The resulting VCF file for each isolate (see “Genomic data processing and variant calling” section above) was compared to the genetic catalogue to determine the presence or absence of resistance-associated mutations for 8 drugs: RIF, INH, EMB, LEV, MXF, AMI, KAN, and ETH. We did not apply the approach used in [8] to make a prediction if a novel mutation was detected in a known resistance gene, as we simply wanted to measure how well a pre-CRyPTIC catalogue could predict resistance in the compendium. These results (found in PREDICTIONS.csv; see “Data availability” section for access) were then compared to the binary phenotypes (see “Binary phenotype classification” section for how these were defined) with the following metrics calculated: TP, the number of phenotypically resistant samples that are correctly identified as resistant (“true positives”); FP, the number of phenotypically susceptible samples that are falsely identified as resistant (“false positives”); TN, the number of phenotypically susceptible samples that are correctly identified as susceptible (“true negatives”); FN, the number of phenotypically resistant samples that are incorrectly identified as susceptible (“false negative”); VME, very major error rate (false-negative rate), 0 to 1; ME, major error rate (false-positive rate), 0 to 1; PPV, positive predictive value, 0 to 1; NPV, negative predictive value.

### Phylogenetic tree construction

A pairwise genetic distance matrix was constructed for 15,211 isolates by comparing pairs of regenotyped VCF files (see “Genomic data processing and variant calling” section above for more details). A neighbourhood-joining tree was constructed from the distance matrix using *quicktree* [53]. Tree visualisation and annotation were performed using the R library *ggtree* [54]. *M*. *tuberculosis* lineages were assigned using Mykrobe and are represented by the coloured dots at the branch termini of the tree [24]. For isolates that had “mixed” lineage classification (i.e., 2 lineages were found present in the sample by Mykrobe, *n =* 225, 1.5%), the first of the 2 lineages was assigned to the isolate. *ggtree* was also used to construct the trees depicting BDQ-, CFZ-, and DLM-resistant isolates.

### The data

Data are available from ftp.ebi.ac.uk/pub/databases/cryptic/release_june2022/.

The FTP site contains 2 top level directories: “reuse” and “reproducibility”. All data for this study were analysed and visualised using either R or python3 libraries and packages. See github.com/kerrimalone/Brankin_Malone_2022 for codebase.

### “reuse” directory

We point the reader to this directory to gain access to CRyPTIC project data. “CRyPTIC_reuse_table_20221019.csv” contains genotypic and phenotypic data relating to the figures and summaries listed in this manuscript and is what we present as a general use reference table for most future projects. It includes binary phenotypes (R/S), MICs, phenotype quality metrics, and ENA sample IDs for 12,288 compendium isolates (see “Quality assurance of the minimum inhibitory concentrations for 13 drugs” section below in Results for filters applied to obtain this final set of isolated). It also includes file paths to each isolate’s VCF file and “regenotyped” VCF file (VCF files that have an entry at all variable positions; see “Genomic data processing and variant calling” section above for more). “CRyPTIC_excluded_samples_20220607.tsv” contains the ENA accession numbers and file paths to each isolate’s VCF file and “regenotyped” VCF file for the 2,922 samples that were sequenced but have no relative MIC data.

### “reproducibility” directory

This directory contains the data used for multiple CRyPTIC project publications referenced throughout this manuscript. As stated above, each project has taken slightly different subsets of these data as documented in those papers. For example, see how tables such as “MUTATIONS.csv” and “GENOTYPES.csv” were used and filtered, (along with others) in this study to obtain the reuse file “CRyPTIC_reuse_table_20221019.csv” in Fig 1. Again, for optimal use of CRyPTIC data in your own project, please refer to “CRyPTIC_reuse_table_20221019.csv” in the “reuse” directory. All data for this study were analysed and visualised using either R or python3 libraries and packages. See github.com/kerrimalone/Brankin_Malone_2022 for codebase.

## Author Contributions

**Conceptualisation:** Daniela M Cirillo, Derrick W. Crook, Philip W Fowler, Sarah Hoosdally, Ana Lúıza Gibertoni Cruz, Nazir A. Ismail, Stefan Niemann, Zamin Iqbal, Tim E.A. Peto, A Sarah Walker, Timothy M Walker

**Data Curation:** Philip W Fowler, Sarah J Hoosdally, Ana Lúıza Gibertoni Cruz, Alice Brankin, Kerri M. Malone, Zamin Iqbal, Martin Hunt and Jeff Knaggs

**Formal Analysis:** Alice Brankin, Kerri M. Malone

**Funding acquisition:** Camilla Rodrigues, David Moore, Derrick W. Crook, Daniela M. Cirillo, Zamin Iqbal, Nazir A. Ismail, Nerges Mistry, Stefan Niemann, Tim E.A. Peto, Guy Thwaites, A. Sarah Walker, Timothy M Walker, Daniel J. Wilson

**Investigation:** Alice Brankin, Kerri M. Malone

**Project Administration:** Daniela M. Cirillo, Derrick W Crook, Philip W Fowler, Sarah Hoosdally, Zamin Iqbal, Tim E.A. Peto, Aysha Roohi,

**Resources:** The CRyPTIC Consortium

**Software:** Philip W Fowler, Martin Hunt, Jeff Knaggs, Brice Letcher

**Supervision:** Daniela M. Cirillo, Derrick W Crook, Philip W Fowler, Zamin Iqbal, Tim E.A. Peto, Daniel J. Wilson

**Validation:** Emanuele Borroni, Daniela M Cirillo, Philip W. Fowler, Clara Grazian, Sarah J. Hoosdally, Martin Hunt, Timothy E. A. Peto, Paola M. V. Rancoita

**Visualisation:** Alice Brankin, Kerri M. Malone

**Writing - original draft preparation**: Alice Brankin, Kerri M. Malone

**Writing - review and editing:** Alice Brankin, Kerri M. Malone, Philip W. Fowler, Zamin Iqbal

## Supporting information

S1 File Supporting information. Text A. Acknowledgements. Text B. Lineages of the *M*. *tuberculosis* isolates of the compendium. Table A. Acronyms used in this manuscript. Table B. Sampling strategies at different collection sites. Table C. Epidemiological cutoff values (ECOFFs) used to binarize MIC measurements into resistant and susceptible. Table D. Lineages–v- geographical location of origin/contribution for CRyPTIC isolates. Table E. Sublineages–v- geographical location of origin/contribution for CRyPTIC isolates. Table F. Sample information for isolates classified as resistant to all 13 drugs tested. Table G. Co-occurrence of antibiotic resistance in CRyPTIC *M*. *tuberculosis* isolates. Supplemental Method A. Generating per-sample and regenotyped VCF files. Fig A. Per-drug MIC distributions of isolates plated on CRyPTIC designed variations on the Thermo Fischer Sensititre MYCOTB MIC plate; UKMYC5 (A) and UKMYC6 (B). Fig B. Geographical distribution of 15,211 CRyPTIC *M*. *tuberculosis* clinical isolates. Fig C. A significant association between country and lineage can be seen in the CRyPTIC data. Fig D. Phylogenetic tree of CRyPTIC *M*. *tuberculosis* clinical isolates. Fig E. Nonsynonymous mutations found outside the RRDR of rpoB in RMR isolates and MDR isolates.
(DOCX)

## Abbreviations

AMI: amikacin
AMR: antimicrobial resistance
AMyGDA: Automated Mycobacterial Growth Detection Algorithm
BDQ: bedaquiline
CFZ: clofazimine
CRyPTIC: Comprehensive Resistance Prediction for Tuberculosis: an International Consortium
CTAB: cetyltrimethylammonium bromide
DLM: delamanid
DR-TB: drug-resistant TB
DST: drug susceptibility testing
ECOFF: epidemiological cutoff
EMB: ethambutol
ETH: ethionamide
FN: false negative
FP: false positive
GCP: genotype confidence percentile
Hr-TB: isoniazid resistant and rifampicin susceptible
INH: isoniazid
KAN: kanamycin
LEV: levofloxacin
LZD: linezolid
MDR: multidrug resistant
ME: major error rate
MIC: minimum inhibitory concentration
MXF: moxifloxacin
NPV: negative predictive value
NRD: new and repurposed drug
PPV: positive predictive value
pre-XDR: pre-extensively drug resistant
RFB: rifabutin
RIF: rifampicin
RRDR: rifampicin resistance–determining region
RMR: rifampicin monoresistant
RR: rifampicin resistant
SNP: single nucleotide polymorphism
TB: tuberculosis
TDR: totally drug resistant
TN: true negative
TP: true positive
VCF: variant call format
VME: very major error rate
WGS: whole-genome sequencing
WHO: World Health Organisation
XDR: extensively drug resistant
