# Supplementary material for: A data compendium associating the genomes of 12,289 Mycobacterium tuberculosis isolates with quantitative resistance phenotypes to 13 antibiotics
Source: PLoS Biol. 2022 Aug 9;20(8):e3001721. doi: 10.1371/journal.pbio.3001721 (PMC9363010; doi:10.1371/journal.pbio.3001721)
Supplement: S1 File — Text A. Acknowledgements. Text B. Lineages of the M. tuberculosis isolates of the compendium. Table A. Acronyms used in this manuscript. Table B. Sampling strategies at different collection sites. Table C. Epidemiological cutoff values (ECOFFs) used to binarize MIC measurements into resistant and susceptible. Table D. Lineages–v- geographical location of origin/contribution for CRyPTIC isolates. Table E. Sublineages–v- geographical location of origin/contribution for CRyPTIC isolates. Table F. Sample information for isolates classified as resistant to all 13 drugs tested. Table G. Co-occurrence of antibiotic resistance in CRyPTIC M. tuberculosis isolates. Supplemental Method A. Generating per-sample and regenotyped VCF files. Fig A. Per-drug MIC distributions of isolates plated on CRyPTIC designed variations on the Thermo Fischer Sensititre MYCOTB MIC plate; UKMYC5 (A) and UKMYC6 (B). Fig B. Geographical distribution of 15,211 CRyPTIC M. tuberculosis clinical isolates. Fig C. A significant association between country and lineage can be seen in the CRyPTIC data. Fig D. Phylogenetic tree of CRyPTIC M. tuberculosis clinical isolates. Fig E. Nonsynonymous mutations found outside the RRDR of rpoB in RMR isolates and MDR isolates. (DOCX) [file pbio.3001721.s001.docx]

**Supplemental Information**

**Text A: Acknowledgements**

The CRyPTIC Consortium Members

Alice Brankin^5,*,**^ and Kerri M Malone^23,*,**^, Ivan Barilar^1^, Simone Battaglia^2^, Emanuele Borroni^2^, Angela Pires Brandao^3,4^, Andrea Maurizio Cabibbe^2^, Joshua Carter^6^, Darren Chetty^7^, Daniela Maria Cirillo^2^, Pauline Claxton^8^, David A Clifton^5^, Ted Cohen^9^, Jorge Coronel^10^, Derrick W Crook^5^, Viola Dreyer^1^, Sarah G Earle^5^, Vincent Escuyer^11^, Lucilaine Ferrazoli^4^, George Fu Gao^12^, Jennifer Gardy^13^, Saheer Gharbia^14^, Kelen Teixeira Ghisi^4^, Arash Ghodousi^2,15^, Ana Lúıza Gibertoni Cruz^5^, Louis Grandjean^16^, Clara Grazian^17^, Ramona Groenheit^18^, Jennifer L Guthrie^19,20^, Wencong He^12^, Harald Hoffmann^21,22^, Sarah J Hoosdally^5^, Martin Hunt^23,5^, Nazir Ahmed Ismail^24^, Lisa Jarrett^25^, Lavania Joseph^24^, Ruwen Jou^26^, Priti Kambli^27^, Rukhsar Khot^27^, Jeff Knaggs^23,5^, Anastasia Koch^28^, Donna Kohlerschmidt^11^, Samaneh Kouchaki^5,29^, Alexander S Lachapelle^5^, Ajit Lalvani^30^, Simon Grandjean Lapierre^31^, Ian F Laurenson^8^, Brice Letcher^23^, Wan-Hsuan Lin^26^, Chunfa Liu^12^, Dongxin Liu^12^, Ayan Mandal^32^, Mikael Mansjo^18^, Daniela Matias^25^, Graeme Meintjes^28^, Flávia de Freitas Mendes^4^, Matthias Merker^1^, Marina Mihalic^22^, James Millard^7^, Paolo Miotto^2^, Nerges Mistry^32^, David Moore^33,10^, Kimberlee A Musser^11^, Dumisani Ngcamu^24^, Hoang Ngoc Nhung^34^, Stefan Niemann^1,35^, Kayzad Soli Nilgiriwala^32^, Camus Nimmo^16^, Max O’Donnell^36^, Nana Okozi^24^, Rosangela Siqueira Oliveira^4^, Shaheed Vally Omar^24^, Nicholas Paton^37^, Timothy EA Peto^5^, Juliana Maira Watanabe Pinhata^4^, Sara Plesnik^22^, Zully M Puyen^38^, Marie Sylvianne Rabodoarivelo^39^, Niaina Rakotosamimanana^39^, Paola MV Rancoita^15^, Priti Rathod^25^, Esther Robinson^25^, Gillian Rodger^5^, Camilla Rodrigues^27^, Timothy C Rodwell^40,41^, Aysha Roohi^5^, David Santos-Lazaro^38^, Sanchi Shah^32^, Thomas Andreas Kohl^1^, Grace Smith^25,14^, Walter Solano^10^, Andrea Spitaleri^2,15^, Philip Supply^42^, Adrie JC Steyn^7^, Utkarsha Surve^27^, Sabira Tahseen^43^, Nguyen Thuy Thuong Thuong^34^, Guy Thwaites^34,5^, Katharina Todt^22^, Alberto Trovato^2^, Christian Utpatel^1^, Annelies Van Rie^44^, Srinivasan Vijay^45^, Timothy M Walker^5,34^, A Sarah Walker^5^, Robin Warren^46^, Jim Werngren^18^, Maria Wijkander^18^, Robert J Wilkinson^47,48,30^, Daniel J Wilson^5^, Penelope Wintringer^23^, Yu-Xin Xiao^26^, Yang Yang^5,50^, Zhao Yanlin^12^, Shen-Yuan Yao^24^, Baoli Zhu^49^, Philip W Fowler^5**^, Zamin Iqbal^23**^

*equal contribution authors
**co-corresponding authors: alice.brankin@ndm.ox.ac.uk, kmalone@ebi.ac.uk, philip.fowler@ndm.ox.ac.uk, zi@ebi.ac.uk

^1^Research Center Borstel, Borstel, Germany

^2^IRCCS San Raffaele Scientific Institute, Milan, Italy

^3^Oswaldo Cruz Foundation, Rio de Janeiro, Brazil

^4^Institute Adolfo Lutz, Sào Paulo, Brazil

^5^University of Oxford, Oxford, UK

^6^Stanford University School of Medicine, Stanford, USA

^7^Africa Health Research Institute, Durban, South Africa

^8^Scottish Mycobacteria Reference Laboratory, Edinburgh, UK

^9^Yale School of Public Health, Yale, USA

^10^Universidad Peruana Cayetano Heredia, Lima, Peru ́

^11^Wadsworth Center, New York State Department of Health, Albany, USA

^12^Chinese Center for Disease Control and Prevention, Beijing, China

^13^Bill & Melinda Gates Foundation, Seattle, USA

^14^UK Health Security Agency, London, UK

^15^Vita-Salute San Raffaele University, Milan, Italy

^16^University College London, London, UK

^17^University of New South Wales, Sydney, Australia

^18^Public Health Agency of Sweden, Solna, Sweden

^19^The University of British Columbia, Vancouver, Canada

^20^Public Health Ontario, Toronto, Canada

^21^SYNLAB Gauting, Munich, Germany

^22^Institute of Microbiology and Laboratory Medicine, IMLred, WHO-SRL Gauting, Germany ^23^EMBL-EBI, Hinxton, UK

^24^National Institute for Communicable Diseases, Johannesburg, South Africa

^25^UK Health Security Agency, Birmingham, UK

^26^Taiwan Centers for Disease Control, Taipei, Taiwan

^27^Hinduja Hospital, Mumbai, India

^28^University of Cape Town, Cape Town, South Africa

^29^University of Surrey, Guildford, UK

^30^Imperial College, London, UK

^31^Université de Montréal, Canada

^32^The Foundation for Medical Research, Mumbai, India

^33^London School of Hygiene and Tropical Medicine, London, UK

^34^Oxford University Clinical Research Unit, Ho Chi Minh City, Viet Nam

^35^German Center for Infection Research (DZIF), Hamburg-Lübeck-Borstel-Riems, Germany ^36^Colombia University Irving Medical Center, New York, USA

^37^National University of Singapore, Singapore

^38^Instituto Nacional de Salud, Lima, Peru ́

^39^Institut Pasteur de Madagascar, Antananarivo, Madagascar

^40^FIND, Geneva, Switzerland

^41^University of California, San Diego, USA

^42^Univ. Lille, CNRS, Inserm, CHU Lille, Institut Pasteur de Lille, U1019 - UMR 9017 - CIIL - Center for Infection and Immunity of Lille, F-59000 Lille, France

^43^National TB Reference Laboratory, National TB Control Program, Islamabad, Pakistan ^44^University of Antwerp, Antwerp, Belgium

^45^University of Edinburgh, Edinburgh, UK

^46^Stellenbosch University, Cape Town, South Africa

^47^Wellcome Centre for Infectious Diseases Research in Africa, Cape Town, South Africa ^48^Francis Crick Institute, London, UK

^49^Institute of Microbiology, Chinese Academy of Sciences, Beijing, China

^50^School of Public Health, Shanghai Jiao Tong University School of Medicine, Shanghai, China. 200025

**Text B:** **Lineages of the *M. tuberculosis* isolates of the compendium.**

Isolates of the ancient Indo-oceanic lineage/L1 (*n* = 1,150) were mostly contributed by India (*n* = 676 isolates) and Vietnam (*n* = 283 isolates). 85% of the L1 Indian isolates belong to sub-lineages 1.1.2 and 1.2.2 while 66% of the Vietnamese isolates are sub-lineage 1.1.1.1. No L1 isolates were contributed by 10 of the 23 countries in this study with only 2 isolates collected in South America (one each in Brazil and Peru).

There are 5,598 L2 (East Asian) isolates, making it the second largest group in the dataset. L2 was found most prevalent in Asia and Europe with the largest proportion found amongst isolates contributed by China (*n* = 722, 64% of isolates) and India (*n* = 1,573, 39% of isolates). Sub-lineages 2.2 and 2.2.7 dominate the L2 isolates (*n* = 1,421 and 1,249 respectively); 2.2 was found mostly amongst Peruvian and South African isolates (*n* = 231 and 161 respectively) apart from those contributed by the Asian countries of Vietnam (*n* = 271), China (*n* = 284) and India (*n* = 272), while 85% of sub-lineage 2.2.7 isolates were contributed by South Africa (*n* = 206), Vietnam (*n* = 164) and India (*n* = 691). 70% of sub-lineage 2.2.1 originated from South Africa (10% of isolates found here) and has recently been associated with lower transmission rates [1]. Lastly, 86% and 72% of isolates contributed by Kyrgyzstan and Turkmenistan respectively belong to L2 with sub-lineage 2.2.10 dominating (16/24 and 75/86 isolates for both countries respectively). 2.2.10 has been previously described as restricted to Central Asia and this is reflected in the compendium [2].

The majority (1184/1850, 65%) of L3 (East African/Indian) isolates were contributed by India, followed by 19.6% (363/1850) isolates from Pakistan. L3 is typically under-sampled and under-studied in current databases and biobanks in comparison to L2 and L4; the L3 isolates in this study are the largest collected to date in a single study [3].

L4 (Euro-American) is the largest lineage group in the compendium (*n* = 6,572). Isolates donated from Peru dominate; 87% of all L4 isolates are Peruvian with 4.1.2.1 and 4.3.3 being the most prevalent sub-lineages (24% and 22% respectively). There are 34 different L4 sub-lineages in the dataset, making L4 the most diverse in comparison to the other lineage groupings.

There were no L5 isolates found in the compendium and only 6 L6 isolates were identified. Animal-restricted pathogenic mycobacterial isolates are also rare in the compendium; only 16 cases were identified (*n* = 15 *M. bovis* and *n* = 1 *M. caprae*).

**Table A: Acronyms used in this manuscript**

| ACRONYM | EXPLANATION |
| --- | --- |
| AMI | Amikacin |
| AMR | Antimicrobial resistance |
| BDQ | Bedaquiline |
| CRyPTIC | Comprehensive Resistance Prediction for Tuberculosis: an International Consortium |
| CFZ | Clofazimine |
| DLM | Delamanid |
| DR-TB | Drug resistant tuberculosis |
| DST | Drug susceptibility testing |
| ECOFF | Epidemiological cut-off |
| EMB | Ethambutol |
| ETH | Ethionamide |
| FN | False negative |
| FP | False positive |
| Hr-TB | Isoniazid resistant and rifampicin susceptible |
| INH | Isoniazid |
| KAN | Kanamycin |
| LEV | Levofloxacin |
| LZD | Linezolid |
| MDR | Multi-drug resistant (resistant to first-line drugs isoniazid and rifampicin) |
| ME | Major error rate |
| MXF | Moxifloxacin |
| NPV | Negative predictive value |
| NRDs | New and repurposed drugs |
| PPV | Positive predictive value |
| pre-XDR | Pre-extensively drug resistant (RR/MDR + fluoroquinolone resistance) |
| RIF | Rifampcin |
| RFB | Rifabutin |
| RMR | Rifampicin mono-resistant |
| RR | Rifampicin resistant |
| RRDR | Rifampicin-resistance determining region |
| SNP | Single nucleotide polymorphism |
| TB | Tuberculosis |
| TDR | Totally drug resistant |
| TN | True negative |
| TP | True positive |
| VME | False negative rate |
| WGS | Whole genome sequencing |
| WHO | World health organisation |
| XDR | Extensively drug resistant (MDR/RR + resistance to at least one fluoroquinolone and either bedaquiline or linezolid) |

**Table B: Sampling strategies at different collection sites**

| Centre | Prospective All | Prospective Enriched R | Freezer All | Freezer enriched R | All NRD R |
| --- | --- | --- | --- | --- | --- |
| China | yes | no | no | no | no |
| Germany | no | yes | no | yes | yes |
| Mumbai | yes | no | no | no | no |
| Peru | yes | no | no | no | no |
| Italy | no | yes | no | yes | no |
| Vietnam | no | yes | no | yes | no |
| NICD | no | yes | yes | no | yes |
| Sweden | no | no | no | yes | no |
| Taiwan | no | yes | no | yes | no |
| Brazil | no | no | no | no | no |
| AHRI | yes | no | no | no | no |
| CapeTown | yes | yes | yes | yes | no |
| Scotland | no | yes | no | yes | no |

*Prospective All* means all culture positive clinical samples were prospectively included in the study. *Prospective Enriched R* means samples were prospectively collected, but selection for inclusion in the project was biassed towards resistant samples. *Freezer All* means retrospective samples were included in the study. *Freezer enriched R* means retrospective samples were selected with a bias towards resistant samples. *All NRD R* means all isolates which were resistant to any of the New and Repurposed Drugs (bedaquiline, clofazimine, delamanid, linezolid) were included. It is possible for a centre to say “yes” for multiple categories, for example if over the period of the project they switched from including all resistant prospective samples to simply including all (resistant or not).

**Table C: Epidemiological cut-off values (ECOFFs) used to binarize MIC measurements into resistant and susceptible.**

| **DRUG** | **ECOFF** |
| --- | --- |
| Isoniazid | 0.1 |
| Rifampicin  Rifabutin  Ethambutol  Ethionamide  Levofloxacin  Moxifloxacin  Amikacin  Kanamycin  Bedaquiline  Clofazimine  Delamanid  Linezolid | 0.5  0.12  4  4  1  1  1  4  0.25  0.25  0.12  1 |

Isolates with an MIC above the cut-off are considered resistant and those at or below the cut-off as susceptible [4].

**Table D: Lineages –v- geographical location of origin/contribution for CRyPTIC isolates**

|  | Animal/other | L1 | L2 | L3 | L4 | L6 | Total |
| --- | --- | --- | --- | --- | --- | --- | --- |
| Albania | 0 | 0 | 0 | 0 | 7 | 0 | 7 |
| Algeria | 0 | 0 | 0 | 0 | 25 | 0 | 25 |
| Brazil | 1 | 1 | 8 | 0 | 335 | 0 | 345 |
| Burkina Faso | 0 | 3 | 1 | 8 | 65 | 1 | 78 |
| China | 7 | 2 | 722 | 16 | 374 | 0 | 1121 |
| Germany | 1 | 25 | 235 | 61 | 403 | 1 | 726 |
| India | 11 | 676 | 1573 | 1184 | 560 | 0 | 4004 |
| Italy | 11 | 38 | 192 | 113 | 866 | 4 | 1224 |
| Japan | 0 | 0 | 0 | 0 | 1 | 0 | 1 |
| Kyrgyzstan | 0 | 0 | 25 | 0 | 3 | 0 | 28 |
| Nepal | 0 | 5 | 137 | 34 | 21 | 0 | 197 |
| Nigeria | 0 | 0 | 0 | 0 | 15 | 0 | 15 |
| Pakistan | 1 | 30 | 23 | 363 | 74 | 0 | 491 |
| Peru | 2 | 1 | 360 | 0 | 2328 | 0 | 2691 |
| South Africa | 1 | 61 | 874 | 57 | 1162 | 0 | 2155 |
| Sweden | 0 | 7 | 280 | 6 | 134 | 0 | 427 |
| Taiwan | 0 | 18 | 149 | 0 | 44 | 0 | 211 |
| Tajikistan | 0 | 0 | 15 | 0 | 4 | 0 | 19 |
| Tunisia | 0 | 0 | 0 | 0 | 2 | 0 | 2 |
| Turkmenistan | 0 | 0 | 88 | 1 | 30 | 0 | 119 |
| Ukraine | 0 | 0 | 21 | 0 | 9 | 0 | 30 |
| UK | 0 | 0 | 3 | 3 | 1 | 0 | 7 |
| Vietnam | 0 | 283 | 892 | 4 | 109 | 0 | 1288 |
| Total | 35 | 1150 | 5598 | 1850 | 6572 | 6 | 15211 |

**Table E: Sub-lineages –v- geographical location of origin/contribution for CRyPTIC isolates**

|  | **ALB** | **ALG** | **BRZ** | **BFA** | **CHN** | **GER** | **IND** | **ITL** | **JPN** | **KGZ** | **NPL** | **NGA** | **PAK** | **PER** | **ZAF** | **SWE** | **TWN** | **TJK** | **TUN** | **TKM** | **UKR** | **UK** | **VNM** |
| --- | --- | --- | --- | --- | --- | --- | --- | --- | --- | --- | --- | --- | --- | --- | --- | --- | --- | --- | --- | --- | --- | --- | --- |
| **1** | 0 | 0 | 0 | 0 | 0 | 0 | 1 | 0 | 0 | 0 | 0 | 0 | 0 | 0 | 0 | 0 | 0 | 0 | 0 | 0 | 0 | 0 | 0 |
| **1.1.1** | 0 | 0 | 0 | 2 | 0 | 0 | 1 | 2 | 0 | 0 | 0 | 0 | 0 | 0 | 0 | 0 | 0 | 0 | 0 | 0 | 0 | 0 | 38 |
| **1.1.1.1** | 0 | 0 | 0 | 0 | 0 | 2 | 0 | 0 | 0 | 0 | 0 | 0 | 0 | 0 | 0 | 0 | 0 | 0 | 0 | 0 | 0 | 0 | 188 |
| **1.1.2** | 0 | 0 | 0 | 1 | 1 | 12 | 316 | 16 | 0 | 0 | 2 | 0 | 23 | 0 | 0 | 1 | 0 | 0 | 0 | 0 | 0 | 0 | 0 |
| **1.1.3** | 0 | 0 | 1 | 0 | 0 | 1 | 52 | 4 | 0 | 0 | 1 | 0 | 1 | 0 | 6 | 0 | 0 | 0 | 0 | 0 | 0 | 0 | 2 |
| **1.2.1** | 0 | 0 | 0 | 0 | 0 | 8 | 12 | 11 | 0 | 0 | 0 | 0 | 0 | 1 | 0 | 3 | 16 | 0 | 0 | 0 | 0 | 0 | 24 |
| **1.2.2** | 0 | 0 | 0 | 0 | 1 | 2 | 259 | 5 | 0 | 0 | 2 | 0 | 4 | 0 | 54 | 3 | 1 | 0 | 0 | 0 | 0 | 0 | 1 |
| **2** | 0 | 0 | 0 | 0 | 0 | 1 | 0 | 0 | 0 | 0 | 0 | 0 | 0 | 0 | 0 | 0 | 0 | 0 | 0 | 0 | 0 | 0 | 0 |
| **2.1** | 0 | 0 | 0 | 0 | 1 | 1 | 0 | 0 | 0 | 0 | 0 | 0 | 0 | 1 | 0 | 0 | 20 | 0 | 0 | 0 | 0 | 0 | 34 |
| **2.2** | 0 | 0 | 5 | 1 | 284 | 19 | 272 | 14 | 0 | 0 | 68 | 0 | 6 | 231 | 181 | 13 | 56 | 0 | 0 | 0 | 0 | 0 | 271 |
| **2.2.1** | 0 | 0 | 0 | 0 | 37 | 4 | 2 | 2 | 0 | 0 | 0 | 0 | 0 | 3 | 224 | 1 | 21 | 0 | 0 | 0 | 0 | 0 | 26 |
| **2.2.10** | 0 | 0 | 0 | 0 | 1 | 117 | 1 | 84 | 0 | 16 | 0 | 0 | 3 | 0 | 0 | 70 | 0 | 5 | 0 | 75 | 9 | 1 | 0 |
| **2.2.2** | 0 | 0 | 0 | 0 | 32 | 8 | 5 | 11 | 0 | 1 | 0 | 0 | 1 | 0 | 0 | 1 | 5 | 0 | 0 | 0 | 1 | 0 | 8 |
| **2.2.3** | 0 | 0 | 2 | 0 | 172 | 3 | 290 | 4 | 0 | 0 | 21 | 0 | 2 | 1 | 3 | 1 | 10 | 0 | 0 | 0 | 0 | 0 | 100 |
| **2.2.4** | 0 | 0 | 0 | 0 | 3 | 3 | 4 | 8 | 0 | 0 | 0 | 0 | 0 | 0 | 37 | 1 | 1 | 0 | 0 | 0 | 0 | 0 | 70 |
| **2.2.5** | 0 | 0 | 0 | 0 | 59 | 1 | 12 | 5 | 0 | 0 | 0 | 0 | 2 | 84 | 1 | 1 | 13 | 0 | 0 | 0 | 0 | 0 | 45 |
| **2.2.6** | 0 | 0 | 1 | 0 | 22 | 8 | 226 | 2 | 0 | 0 | 9 | 0 | 7 | 4 | 204 | 1 | 4 | 0 | 0 | 0 | 0 | 0 | 154 |
| **2.2.7** | 0 | 0 | 0 | 0 | 97 | 4 | 691 | 5 | 0 | 1 | 38 | 0 | 0 | 20 | 206 | 5 | 18 | 0 | 0 | 0 | 0 | 0 | 164 |
| **2.2.8** | 0 | 0 | 0 | 0 | 4 | 0 | 42 | 0 | 0 | 0 | 1 | 0 | 2 | 0 | 0 | 0 | 0 | 0 | 0 | 0 | 0 | 0 | 2 |
| **2.2.9** | 0 | 0 | 0 | 0 | 0 | 64 | 0 | 51 | 0 | 6 | 0 | 0 | 0 | 2 | 0 | 174 | 0 | 10 | 0 | 11 | 11 | 2 | 0 |
| **3** | 0 | 0 | 0 | 8 | 15 | 37 | 828 | 81 | 0 | 0 | 28 | 0 | 346 | 0 | 34 | 5 | 0 | 0 | 0 | 1 | 0 | 2 | 2 |
| **3.1.1** | 0 | 0 | 0 | 0 | 0 | 17 | 17 | 25 | 0 | 0 | 1 | 0 | 0 | 0 | 19 | 1 | 0 | 0 | 0 | 0 | 0 | 1 | 2 |
| **3.1.2** | 0 | 0 | 0 | 0 | 1 | 5 | 201 | 3 | 0 | 0 | 1 | 0 | 7 | 0 | 2 | 0 | 0 | 0 | 0 | 0 | 0 | 0 | 0 |
| **3.1.2.1** | 0 | 0 | 0 | 0 | 0 | 2 | 119 | 3 | 0 | 0 | 4 | 0 | 6 | 0 | 0 | 0 | 0 | 0 | 0 | 0 | 0 | 0 | 0 |
| **4** | 0 | 0 | 1 | 0 | 6 | 4 | 0 | 6 | 0 | 0 | 0 | 0 | 0 | 100 | 1 | 1 | 0 | 0 | 0 | 1 | 0 | 0 | 0 |
| **4.1** | 0 | 0 | 0 | 1 | 0 | 1 | 0 | 2 | 0 | 0 | 0 | 0 | 0 | 0 | 0 | 0 | 0 | 0 | 0 | 0 | 0 | 0 | 0 |
| **4.1.1** | 0 | 0 | 0 | 0 | 0 | 6 | 5 | 10 | 0 | 0 | 1 | 0 | 0 | 154 | 13 | 1 | 0 | 0 | 0 | 0 | 0 | 0 | 0 |
| **4.1.1.1** | 0 | 0 | 5 | 0 | 1 | 3 | 20 | 5 | 0 | 0 | 1 | 0 | 2 | 5 | 54 | 1 | 0 | 0 | 0 | 0 | 0 | 0 | 1 |
| **4.1.1.2** | 0 | 0 | 0 | 0 | 0 | 0 | 4 | 1 | 0 | 0 | 0 | 0 | 0 | 0 | 6 | 0 | 0 | 0 | 0 | 0 | 0 | 0 | 0 |
| **4.1.1.3** | 0 | 0 | 1 | 6 | 1 | 0 | 30 | 7 | 1 | 0 | 3 | 0 | 2 | 68 | 138 | 0 | 0 | 0 | 0 | 0 | 0 | 0 | 1 |
| **4.1.2** | 0 | 0 | 13 | 0 | 0 | 15 | 3 | 26 | 0 | 1 | 0 | 0 | 0 | 31 | 43 | 0 | 0 | 0 | 0 | 0 | 0 | 0 | 10 |
| **4.1.2.1** | 1 | 8 | 87 | 10 | 0 | 111 | 56 | 183 | 0 | 0 | 3 | 2 | 4 | 653 | 54 | 5 | 0 | 1 | 0 | 0 | 1 | 0 | 17 |
| **4.1.3** | 0 | 0 | 0 | 5 | 0 | 0 | 0 | 6 | 0 | 0 | 0 | 1 | 0 | 0 | 0 | 0 | 0 | 0 | 0 | 0 | 0 | 0 | 0 |
| **4.10** | 4 | 6 | 35 | 5 | 1 | 110 | 166 | 207 | 0 | 1 | 3 | 0 | 28 | 128 | 170 | 4 | 5 | 2 | 1 | 6 | 2 | 0 | 12 |
| **4.2** | 0 | 0 | 0 | 0 | 3 | 1 | 0 | 1 | 0 | 0 | 0 | 0 | 0 | 0 | 0 | 0 | 0 | 0 | 0 | 0 | 0 | 0 | 0 |
| **4.2.1** | 0 | 0 | 0 | 0 | 0 | 9 | 3 | 46 | 0 | 0 | 1 | 0 | 2 | 0 | 2 | 4 | 0 | 0 | 0 | 8 | 3 | 1 | 0 |
| **4.2.2** | 0 | 1 | 0 | 0 | 53 | 31 | 76 | 51 | 0 | 0 | 5 | 0 | 12 | 2 | 10 | 10 | 1 | 0 | 0 | 0 | 0 | 0 | 11 |
| **4.2.2.1** | 0 | 0 | 0 | 0 | 0 | 5 | 0 | 9 | 0 | 0 | 0 | 0 | 0 | 0 | 0 | 0 | 0 | 0 | 0 | 0 | 0 | 0 | 0 |
| **4.3** | 0 | 0 | 17 | 0 | 0 | 0 | 0 | 1 | 0 | 0 | 0 | 0 | 0 | 96 | 1 | 0 | 0 | 0 | 0 | 0 | 0 | 0 | 0 |
| **4.3.1** | 0 | 3 | 0 | 1 | 0 | 1 | 2 | 21 | 0 | 0 | 0 | 0 | 0 | 8 | 0 | 1 | 0 | 0 | 0 | 0 | 0 | 0 | 1 |
| **4.3.2** | 0 | 1 | 10 | 0 | 0 | 3 | 1 | 8 | 0 | 0 | 0 | 0 | 0 | 245 | 21 | 0 | 0 | 0 | 0 | 0 | 0 | 0 | 0 |
| **4.3.2.1** | 0 | 0 | 0 | 0 | 0 | 0 | 0 | 0 | 0 | 0 | 0 | 0 | 0 | 0 | 234 | 0 | 0 | 0 | 0 | 0 | 0 | 0 | 0 |
| **4.3.3** | 1 | 4 | 34 | 2 | 0 | 33 | 99 | 81 | 0 | 1 | 1 | 0 | 0 | 576 | 120 | 102 | 2 | 1 | 1 | 11 | 2 | 0 | 1 |
| **4.3.4** | 0 | 0 | 1 | 0 | 0 | 0 | 8 | 0 | 0 | 0 | 0 | 0 | 0 | 1 | 0 | 0 | 0 | 0 | 0 | 0 | 0 | 0 | 0 |
| **4.3.4.1** | 0 | 0 | 70 | 0 | 0 | 2 | 6 | 3 | 0 | 0 | 0 | 0 | 0 | 60 | 22 | 2 | 1 | 0 | 0 | 0 | 0 | 0 | 0 |
| **4.3.4.2** | 0 | 0 | 38 | 0 | 0 | 6 | 7 | 27 | 0 | 0 | 0 | 0 | 0 | 132 | 12 | 0 | 0 | 0 | 0 | 0 | 1 | 0 | 0 |
| **4.3.4.2.1** | 0 | 0 | 0 | 0 | 0 | 2 | 1 | 1 | 0 | 0 | 0 | 0 | 0 | 0 | 75 | 0 | 0 | 0 | 0 | 0 | 0 | 0 | 0 |
| **4.4** | 0 | 0 | 0 | 0 | 0 | 0 | 13 | 0 | 0 | 0 | 0 | 0 | 0 | 1 | 0 | 0 | 0 | 0 | 0 | 0 | 0 | 0 | 0 |
| **4.4.1** | 0 | 0 | 10 | 0 | 0 | 0 | 0 | 2 | 0 | 0 | 0 | 0 | 0 | 11 | 0 | 0 | 0 | 0 | 0 | 0 | 0 | 0 | 0 |
| **4.4.1.1** | 0 | 0 | 4 | 0 | 0 | 16 | 4 | 66 | 0 | 0 | 1 | 0 | 0 | 22 | 180 | 2 | 0 | 0 | 0 | 0 | 0 | 0 | 4 |
| **4.4.1.2** | 0 | 0 | 0 | 0 | 0 | 1 | 25 | 3 | 0 | 0 | 0 | 0 | 0 | 2 | 0 | 0 | 0 | 0 | 0 | 0 | 0 | 0 | 3 |
| **4.4.2** | 0 | 0 | 0 | 0 | 124 | 1 | 4 | 1 | 0 | 0 | 0 | 0 | 0 | 4 | 0 | 0 | 5 | 0 | 0 | 0 | 0 | 0 | 16 |
| **4.5** | 0 | 0 | 0 | 0 | 179 | 28 | 15 | 14 | 0 | 0 | 1 | 0 | 18 | 5 | 1 | 0 | 30 | 0 | 0 | 3 | 0 | 0 | 29 |
| **4.6** | 0 | 1 | 0 | 1 | 0 | 1 | 1 | 7 | 0 | 0 | 0 | 2 | 0 | 0 | 0 | 0 | 0 | 0 | 0 | 0 | 0 | 0 | 0 |
| **4.6.1.1** | 0 | 0 | 0 | 0 | 0 | 1 | 0 | 0 | 0 | 0 | 0 | 0 | 0 | 0 | 0 | 0 | 0 | 0 | 0 | 0 | 0 | 0 | 0 |
| **4.6.1.2** | 0 | 0 | 0 | 0 | 0 | 1 | 0 | 0 | 0 | 0 | 0 | 0 | 0 | 0 | 0 | 1 | 0 | 0 | 0 | 0 | 0 | 0 | 0 |
| **4.6.2** | 0 | 0 | 0 | 0 | 0 | 2 | 2 | 14 | 0 | 0 | 0 | 0 | 4 | 0 | 0 | 0 | 0 | 0 | 0 | 0 | 0 | 0 | 0 |
| **4.6.2.1** | 0 | 0 | 0 | 0 | 0 | 0 | 1 | 0 | 0 | 0 | 1 | 0 | 1 | 0 | 0 | 0 | 0 | 0 | 0 | 0 | 0 | 0 | 0 |
| **4.6.2.2** | 1 | 0 | 0 | 34 | 0 | 8 | 1 | 55 | 0 | 0 | 0 | 10 | 1 | 0 | 1 | 0 | 0 | 0 | 0 | 0 | 0 | 0 | 3 |
| **6** | 0 | 0 | 0 | 1 | 0 | 1 | 0 | 4 | 0 | 0 | 0 | 0 | 0 | 0 | 0 | 0 | 0 | 0 | 0 | 0 | 0 | 0 | 0 |
| **Bovis** | 0 | 0 | 0 | 0 | 0 | 1 | 1 | 10 | 0 | 0 | 0 | 0 | 0 | 2 | 1 | 0 | 0 | 0 | 0 | 0 | 0 | 0 | 0 |
| **Caprae** | 0 | 0 | 0 | 0 | 0 | 0 | 0 | 1 | 0 | 0 | 0 | 0 | 0 | 0 | 0 | 0 | 0 | 0 | 0 | 0 | 0 | 0 | 0 |
| **Mixed** | 0 | 1 | 9 | 0 | 16 | 3 | 89 | 9 | 0 | 1 | 0 | 0 | 6 | 38 | 25 | 12 | 2 | 0 | 0 | 3 | 0 | 0 | 48 |
| **Unknown** | 0 | 0 | 1 | 0 | 7 | 0 | 0 | 0 | 0 | 0 | 0 | 0 | 1 | 0 | 0 | 0 | 0 | 0 | 0 | 0 | 0 | 0 | 0 |

**Table F: Sample information for isolates classified as resistant to all 13 drugs tested**.

| UNIQUEID | COUNTRY OF ORIGIN | LINEAGE |
| --- | --- | --- |
| site.11.subj.XTB-18-224.lab.XTB-18-224.iso.1 | UNKNOWN | Lineage 2 |
| site.10.subj.YA00026182.lab.YA00026182.iso.1 | S. Africa | Lineage 4 |

**Table G: Co-occurrence of antibiotic resistance in CRyPTIC *M. tuberculosis* isolates.**

|  | INH | RIF | EMB | LEV | MXF | AMI | KAN | BDQ | CFZ | DLM | LZD | ETH | RFB |
| --- | --- | --- | --- | --- | --- | --- | --- | --- | --- | --- | --- | --- | --- |
| INH | 100.0 | 74.8 | 38.0 | 34.0 | 27.8 | 14.0 | 17.0 | 1.5 | 5.5 | 1.7 | 2.0 | 28.2 | 70.4 |
| RIF | 93.5 | 100.0 | 46.3 | 41.4 | 34.1 | 17.2 | 20.8 | 1.8 | 6.0 | 1.7 | 2.3 | 29.4 | 91.3 |
| EMB | 98.5 | 95.9 | 100.0 | 53.9 | 47.1 | 23.3 | 26.4 | 2.4 | 8.2 | 2.3 | 3.7 | 35.8 | 87.3 |
| LEV | 93.3 | 90.2 | 56.9 | 100.0 | 78.5 | 27.3 | 31.3 | 3.1 | 9.4 | 2.9 | 4.7 | 39.2 | 87.1 |
| MXF | 95.0 | 92.3 | 61.9 | 97.6 | 100.0 | 29.9 | 34.6 | 3.2 | 9.9 | 2.7 | 5.3 | 41.9 | 88.8 |
| AMI | 93.0 | 90.4 | 58.9 | 65.3 | 57.8 | 100.0 | 90.4 | 2.4 | 13.0 | 5.2 | 8.1 | 40.7 | 82.8 |
| KAN | 89.4 | 86.7 | 53.0 | 59.5 | 53.2 | 72.0 | 100.0 | 1.8 | 10.6 | 3.2 | 5.7 | 40.4 | 80.0 |
| BDQ | 79.4 | 77.8 | 49.5 | 60.7 | 49.5 | 20.0 | 18.9 | 100.0 | 52.4 | 12.9 | 14.0 | 34.9 | 76.9 |
| CFZ | 61.9 | 52.8 | 34.9 | 38.2 | 32.1 | 21.8 | 22.5 | 10.6 | 100.0 | 9.4 | 10.2 | 27.0 | 53.7 |
| DLM | 55.2 | 44.4 | 28.2 | 33.2 | 23.9 | 24.4 | 19.1 | 7.1 | 26.3 | 100.0 | 19.3 | 22.1 | 45.4 |
| LZD | 77.6 | 69.9 | 53.2 | 64.7 | 58.7 | 46.7 | 41.2 | 9.8 | 34.2 | 24.1 | 100.0 | 42.6 | 66.7 |
| ETH | 96.5 | 79.4 | 47.0 | 48.7 | 41.9 | 21.1 | 26.2 | 2.2 | 8.3 | 2.4 | 3.9 | 100.0 | 77.1 |
| RFB | 93.3 | 96.8 | 44.5 | 42.1 | 34.4 | 16.6 | 20.3 | 1.9 | 6.4 | 1.9 | 2.3 | 29.9 | 100.0 |

The probability (%) of an isolate being resistant to Drug 2 (top) if it is resistant to Drug 1 (left). Drug acronyms: INH = isoniazid, RIF = rifampicin, EMB = ethambutol, LEV = levofloxacin, MXF = moxifloxacin, AMI = amikacin, KAN = kanamycin, BDQ = bedaquiline, CFZ = clofazimine, DLM = delamanid, LZD = linezolid, ETH = ethionamide, RFB = rifabutin.

**Supplemental Method A: Generating per-sample and regenotyped VCF files**The following commands reproduce the per-sample (steps 1-5) and regenotyped (step 6) VCF files for each sample of the data compendium. As a reminder, the per-sample VCFs are “normal” VCFs which have one record for each variant where the sample differs from the reference. For *M. tuberculosis* these tend to have around 1000-2000 variants, and this number varies for each sample. The regenotyped (or jointly genotyped) VCFs contain one record for every variant found in the whole cohort – thus all samples have exactly the same numbers of records.

There are two code repositories needed: <https://github.com/iqbal-lab-org/clockwork> (steps 1-5, we used v0.8.3) and <https://github.com/iqbal-lab-org/minos> (step 6, we used v0.11.0). To ensure reproducibility and simplify install issues, we provide a singularity container for Clockwork (clockwork_container.v0.8.3.img) in the FTP site. It is also necessary to clone (*i.e.* get) both the Clockwork and minos code repositories in order to run the nextflow scripts below. Commands are:

1. Get decontamination reference file:

singularity exec clockwork_container.img \

/clockwork/scripts/download_tb_reference_files.pl ref_data

1. Make all the reference indexes *etc*. and register it with clockwork:

singularity exec clockwork_container.img clockwork reference_prepare \

--db_config_file db.ini --pipeline_references_root Pipeline_refs \

--contam_tsv ref_data/remove_contam.tsv \

--name remove_contam \

ref_data/remove_contam.fa.gz

1. Add the *Mycobacterium tuberculosis* H37Rv reference genome sequence (NC000962.3):

singularity exec clockwork_container.img clockwork reference_prepare \

--db_config_file db.ini --pipeline_references_root Pipeline_refs \

--name NC_000962.3 \

ref_data/NC_000962.3.fa

1. Remove contaminated reads:

nextflow run nextflow/remove_contam.nf \

-with-singularity clockwork_container.img \

--ref_id 1 \

--references_root Pipeline_refs \

--pipeline_root Pipeline_root \

--db_config_file db.ini

1. Per-sample Variant call:

nextflow run nextflow/variant_call.nf \

-with-singularity clockwork_container.img \

--ref_id 2 \

--references_root Pipeline_refs \

--pipeline_root Pipeline_root \

--db_config_file db.ini

1. Joint genotype, uses a nextflow script from the minos repository:

nextflow run \

-c nextflow/config.nf \

-profile large \

nextflow/regenotype.nf \

--ref_fasta NC_000962.3.fa \

--manifest manifest.tsv \

--outdir <PATH/TO/OUTPUT/DIRECTORY>

**References**

1. Holt KE, McAdam P, Thai PVK, Thuong NTT, Ha DTM, Lan NN, et al. Frequent transmission of the Mycobacterium tuberculosis Beijing lineage and positive selection for the EsxW Beijing variant in Vietnam. Nature Genetics. 2018;50: 849–856. doi:10.1038/s41588-018-0117-9

2. Chiner-Oms Á, Comas I. Large genomics datasets shed light on the evolution of the Mycobacterium tuberculosis complex. Infection, Genetics and Evolution. 2019;72: 10–15. doi:10.1016/j.meegid.2019.02.028

3. Freschi L, Vargas R, Husain A, Kamal SMM, Skrahina A, Tahseen S, et al. Population structure, biogeography and transmissibility of Mycobacterium tuberculosis. Nature Communications. 2021;12: 6099. doi:10.1038/s41467-021-26248-1

4. The CRyPTIC Consortium. Epidemiological cutoff values for a 96-well broth microdilution plate for high-throughput research antibiotic susceptibility testing of M. tuberculosis. European Respiratory Journal. 2022; 2200239. doi:10.1183/13993003.00239-2022

5. Howe K, Bateman A, Durbin R. QuickTree: building huge Neighbour-Joining trees of protein sequences. Bioinformatics. 2002;18: 1546–1547. doi:10.1093/bioinformatics/18.11.1546

6. Borrell S, Trauner A, Brites D, Rigouts L, Loiseau C, Coscolla M, et al. Reference set of Mycobacterium tuberculosis clinical strains: A tool for research and product development. PLOS ONE. 2019;14: e0214088. doi:10.1371/journal.pone.0214088


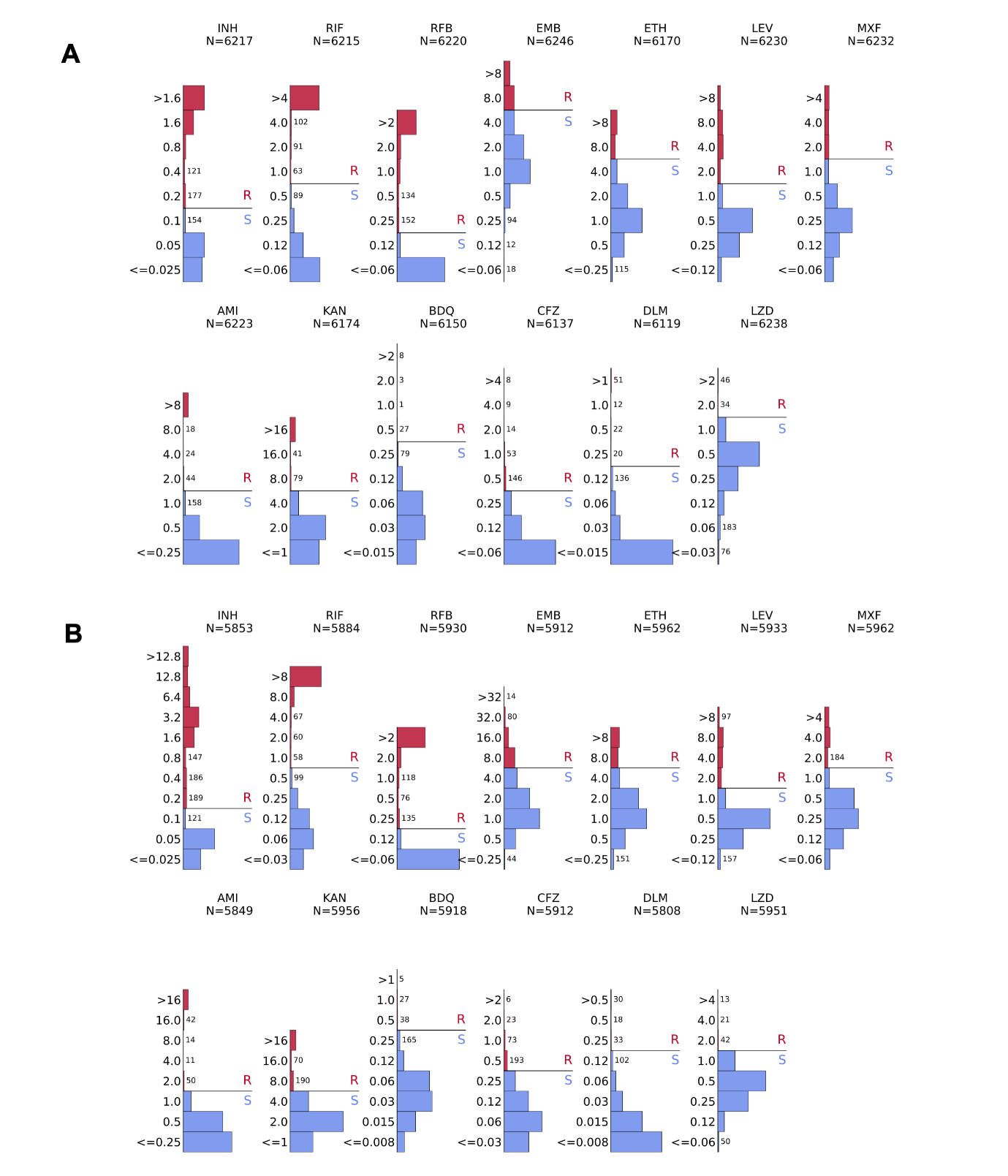


**Fig A: Per drug MIC distributions of isolates plated on CRyPTIC designed variations on the Thermo Fischer Sensititre MYCOTB MIC plate; UKMYC5 (A) and UKMYC6 (B).** The solid black line represents the epidemiological cut-off (ECOFF) values for each drug as determined by [4]. Isolates with an MIC above the cut-off are considered resistant. N denotes the total number of isolates tested on each plate that returned a phenotype for each drug.


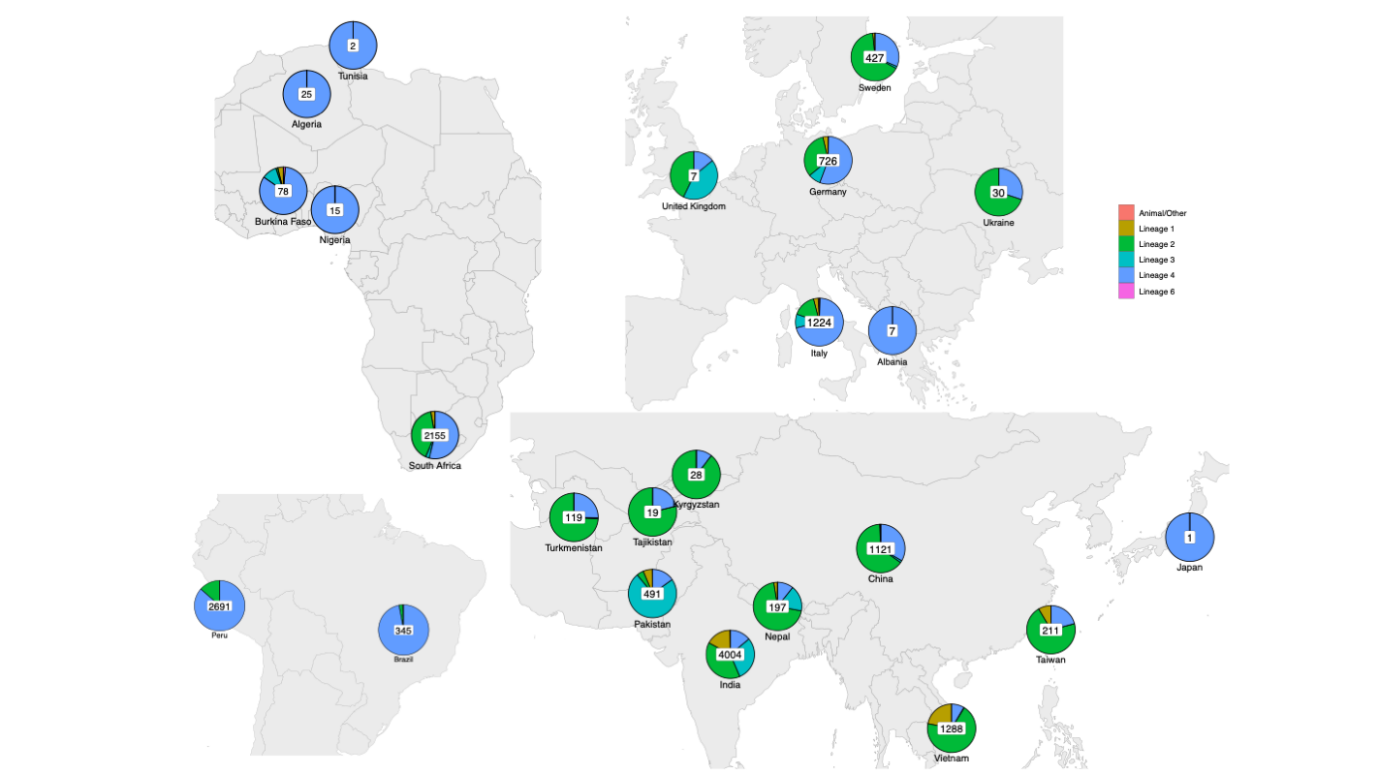


**Fig B: Geographical distribution of 15,211 CRyPTIC *M. tuberculosis* clinical isolates.** The total number of isolates contributed by each country is depicted, with pie charts representing the proportion of *M. tuberculosis* lineages. Where the origin of an isolate was not known, the collection site identity was assigned (269 isolates in Germany, 17 isolates in India, 6 isolates in Peru, 885 isolates in Italy, 510 isolates in South Africa, 357 isolates in Sweden, 208 isolates in Taiwan, 1 isolate in Brazil and 4 isolates in the UK).


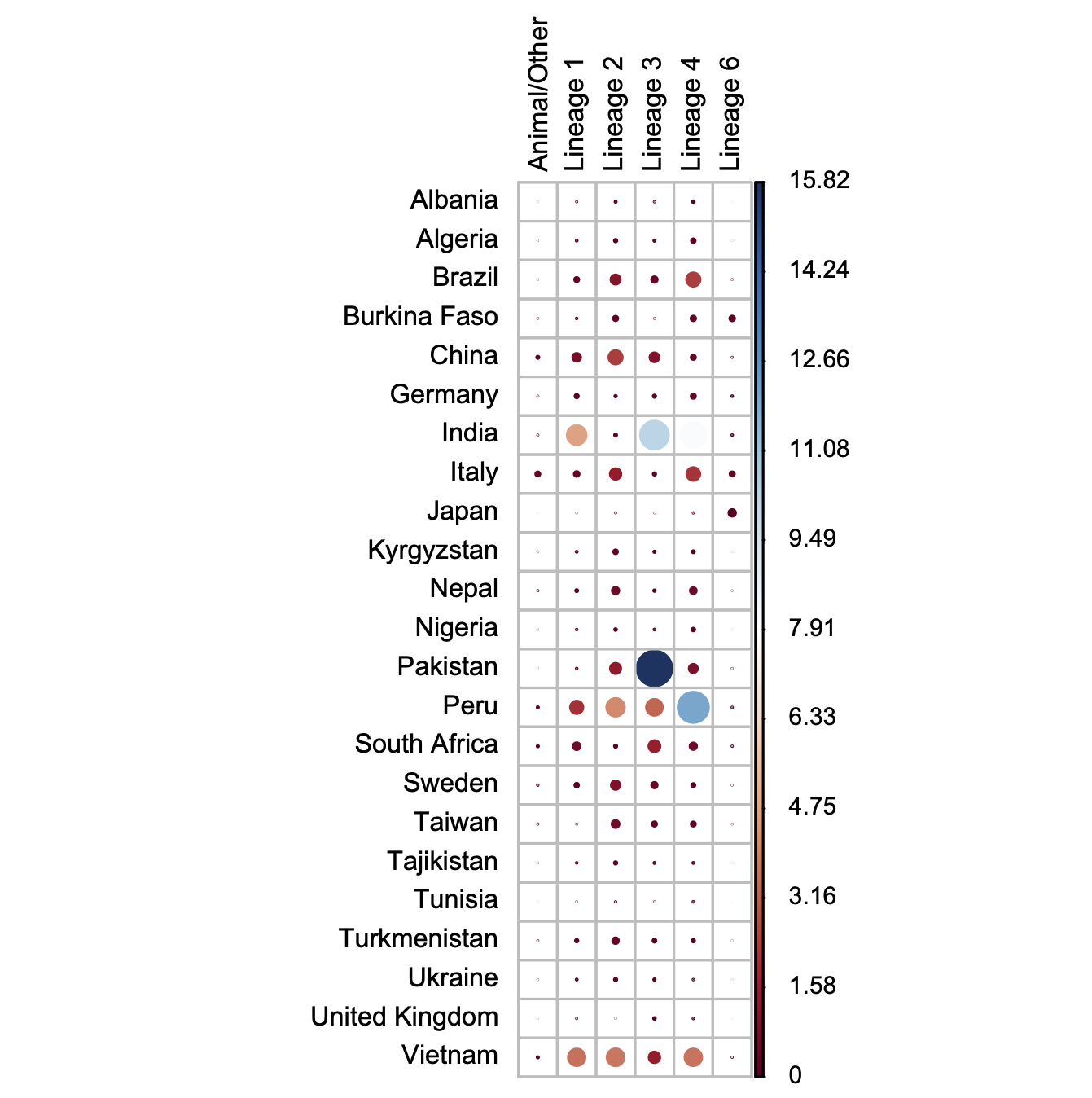


**Fig C**: **A significant association between country and lineage can be seen in the CRyPTIC data.** Pearson’s chi-squared test, X-squared = 7935.2, df = 110, *p* < 2.2e-16. The correlation plot indicates the relative contribution of each row-column pairing to the chi-square test score (%).

**
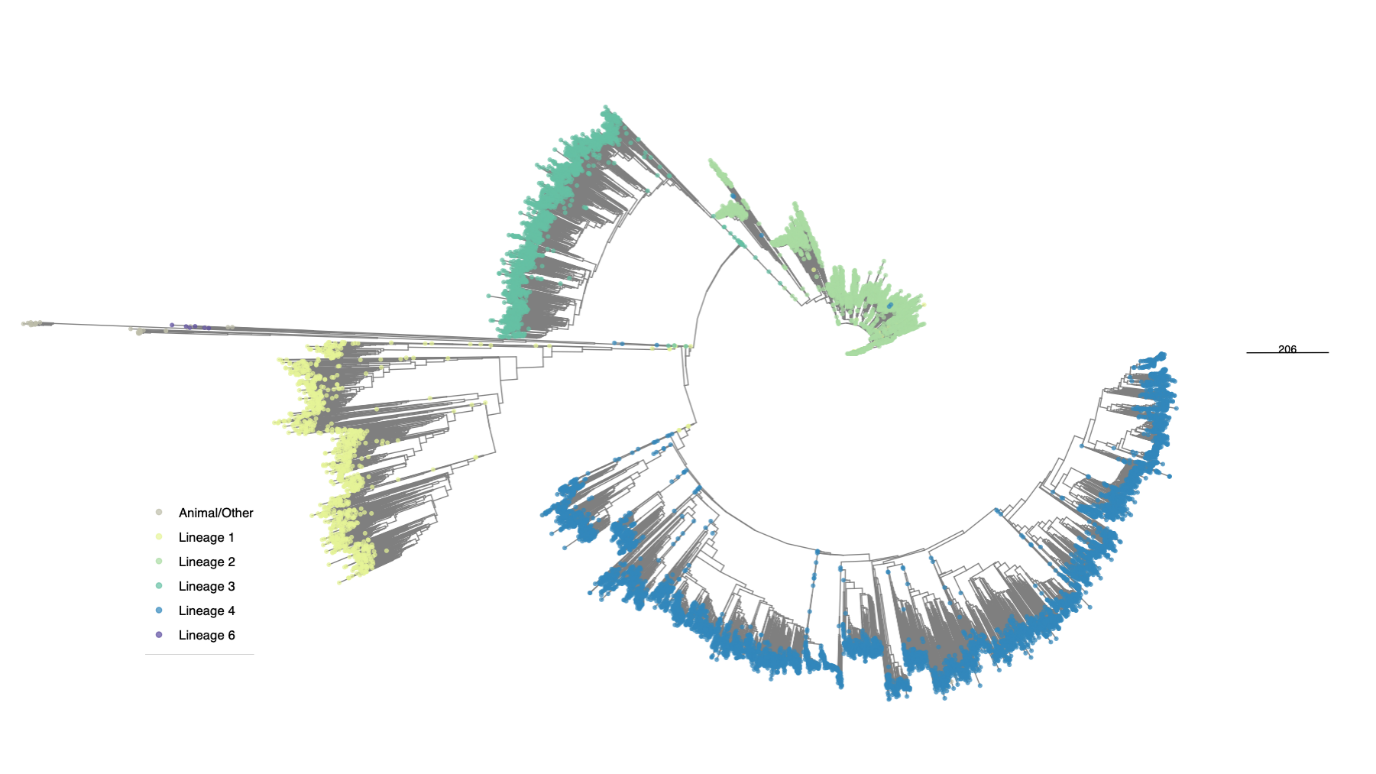
**

**Fig D: Phylogenetic tree of CRyPTIC *M. tuberculosis* clinical isolates.** A phylogenetic cladogram of 15,211 *M. tuberculosis* clinical isolates (“full dataset”). A neighbour-joining tree was constructed from a pairwise distance matrix using *quicktree* [5]. Coloured dots at the branch termini represent the lineage assigned to each isolate. “Animal/Other” includes 16 isolates that were assigned the following lineages: *M. caprae* (1)*, M. bovis* (1)*,* along with 17 isolates previously defined as representative for specific sub-lineages [6].


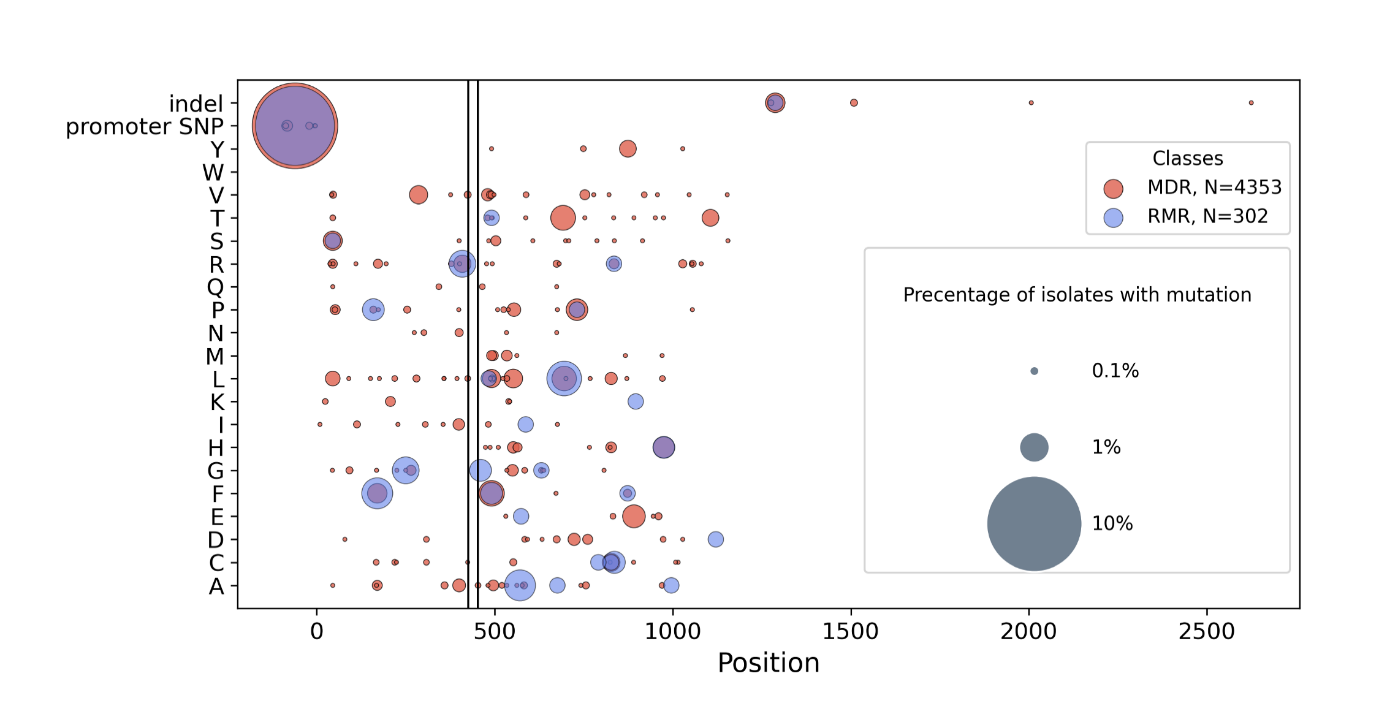


**Fig E: Non-synonymous mutations found outside the RRDR of rpoB in RMR isolates and MDR isolates.** Presence of a coloured spot indicates that the mutation was found in RMR/MDR isolates and spot size corresponds to the proportion of RMR or MDR isolates carrying that mutation.
